# Advances in Living Cell-Mediated Nanodrug Delivery Systems: Construction Strategies, Applications and Challenges

**DOI:** 10.3390/pharmaceutics18091070

**Published:** 2026-08-27

**Authors:** Huaying Pan, Juan Yang, Jiaqi Fu, Shenao Yan, Yue Yan, Yining Xu, Minglu Zhou, Lian Li, Yucheng Xiang

**Affiliations:** 1 Affiliated Hospital of North Sichuan Medical College, Nanchong 637000, China; 2 Key Laboratory of Drug-Targeting and Drug Delivery System of the Education Ministry and Sichuan Province, Sichuan Engineering Laboratory for Plant-Sourced Drug, West China School of Pharmacy, Sichuan University, Chengdu 610041, China; 3 Department of Pharmacy, West China Hospital, Sichuan University, Chengdu 610041, Chinazzhouminglu@163.com (M.Z.); 4 Sichuan Higher Education Institute Key Laboratory of Structure-Specific Small Molecule Drugs, Institute of Materia Medica, School of Pharmacy, Chengdu Medical College, Chengdu 610500, China

**Keywords:** living cell, nanodrug delivery system, targeted therapy, cell–nanocarrier conjugate system

## Abstract

Nanodrug delivery systems have shown great promise against tumors, inflammatory diseases and central nervous system disorders. However, several limitations still exist before further clinical application. The unsatisfactory circulation time in vivo, rapid clearance by the mononuclear phagocyte system, insufficient tissue penetration and low targeting efficiency all hinder the clinical translation of nanomedicines. Cells are the fundamental functional units of the body and their intrinsic biological properties make them promising drug delivery vehicles for targeted therapy. Conjugating cells with nanomedicines combines the carrier functions of living cells with the therapeutic effects of nanodrugs, offering an effective strategy to improve targeted drug delivery. Compared with the existing literature, this review specifically summarizes the biological properties and major applications of six types of living cell carriers. The recent strategies for constructing cell–nanomedicine conjugates are also discussed. Finally, we highlight the current challenges for clinical translation and discuss future directions for the development of cell–nanomedicine delivery systems.

## 1. Introduction

Cancer, inflammatory diseases and central nervous system (CNS) disorders have become major global public health challenges [1,2,3,4]. Cancer remains one of the leading causes of mortality worldwide, while conventional chemotherapy and radiotherapy are impeded by severe systemic toxicity, drug resistance and high recurrence rates. Inflammatory diseases, such as rheumatoid arthritis and systemic lupus erythematosus, often exhibit chronic progression. The long-term immunosuppressive therapy tends to cause infections and organ damage in patients. Meanwhile, the treatment of CNS disorders, including Alzheimer’s disease, Parkinson’s disease and glioblastoma, is largely restricted by the blood–brain barrier (BBB) and poor neural tissue regeneration [5,6,7]. In recent years, efficient delivery systems based on nanocarriers (NCs) have shown therapeutic potential in various diseases [8,9,10,11,12,13]. For instance, liposomal doxorubicin substantially reduces the cardiotoxicity of anthracyclines and is successfully used in cancer therapy [14]. Albumin-bound paclitaxel nanoparticles improve drug accumulation in solid tumors [15]. In addition, lipid nanoparticles (LNPs) delivering small interfering RNA (siRNA) (Patisiran) have been approved for the treatment of transthyretin amyloidosis [16]. Furthermore, delivery vehicles such as nanoparticles and exosomes have been extensively investigated for neurological disorders and brain tumors, highlighting their considerable potential [17,18].

Although NCs such as nanoparticles, micelles and liposomes have achieved substantial progress in improving drug solubility, oral bioavailability, reducing toxicity and prolonging circulation time, their application of these vehicles in vivo still faces numerous bottlenecks [19,20,21,22,23,24,25,26]. Firstly, nanomedicines are rapidly cleared by the mononuclear phagocyte system (MPS), resulting in an extremely low fraction reaching the target site, with an average delivering efficiency of less than 1%. Secondly, the widespread enhanced permeability and retention (EPR) effect exhibits significant heterogeneity and interindividual variability in human tumors, thereby limiting clinical delivery efficiency. The dense extracellular matrix and high interstitial pressure restrict deep penetration of nanomedicines, whereas inefficient cellular uptake and inadequate endosomal escape further compromise intracellular delivery [27]. Moreover, some nanomaterials are susceptible to potential toxicity, immune clearance and poor stability, which severely restrict their clinical translation.

To circumvent these obstacles, biomimetic delivery systems such as membrane-coated nanoparticles have been developed, combining the advantages of biological membranes and nanocarriers [28,29,30]. Zhang et al. designed membrane-coated nanoparticles hybridized from erythrocytes and tumor cells, achieving prolonged circulation time and dual functionality for recognizing primary, circulating and metastatic tumors [31]. Although membrane-coated nanoparticles exhibit certain advantages over conventional nanocarriers, they suffer from insufficient structural stability [32,33,34]. On the one hand, the weak adhesion between the cell membrane and the nanocarrier core might result into the detachment of outer coating under the imbalance of blood shear stress and membrane bending stress. On the other hand, functional membrane proteins are prone to denaturation or inactivation during membrane extraction and upon entering the bloodstream, they are easily shielded by the protein corona, thereby failing to mimic the original functions of the cell [35,36,37].

Living cell-based nanodrug delivery systems (LCNDDs) have emerged as a promising drug delivery strategy. Based on the recent studies, the general definition of cellular hitchhiking refers to an unconventional mode of intracellular transport, documented across fungal, plant and animal taxa [38,39]. It relies on a moving primary carrier to convoy a secondary cargo, thereby affecting co-transport. As a result, the accompanying payload is perceived as an endogenous constituent during circulation, allowing it to escape immune or clearance mechanisms [40]. Two major approaches exist. One is intracellular loading via phagocytosis or physical methods. The other is surface attachment via covalent or non-covalent binding. The performance of the “hitchhiking” system largely depends on the inherent properties of the carrier cells. The red blood cells support long circulation. Immune cells actively migrate to inflammatory sites. Stem cells contribute to tissue repair.

This review systematically integrates and critically analyzes recent advances in LCNDDs. We provide a comprehensive analysis of six cell types, including erythrocytes, T cells and NK cells, elaborating on their unique biological properties, functional characteristics and therapeutic applications. Subsequently, we focus on strategies to engineer LCNDDs, including intracellular loading, non-covalent conjugation and covalent conjugation. Finally, we discuss the therapeutic potential of LCNDDs in cancer therapy, immune regulation and CNS disorders, providing perspectives on next-generation living cell carriers. Collectively, LCNDDs hold great promise for overcoming longstanding barriers in drug delivery and advancing transformative therapies for major human diseases.

## 2. Cell Types for Living Cell Drug Delivery

**Figure 1:**
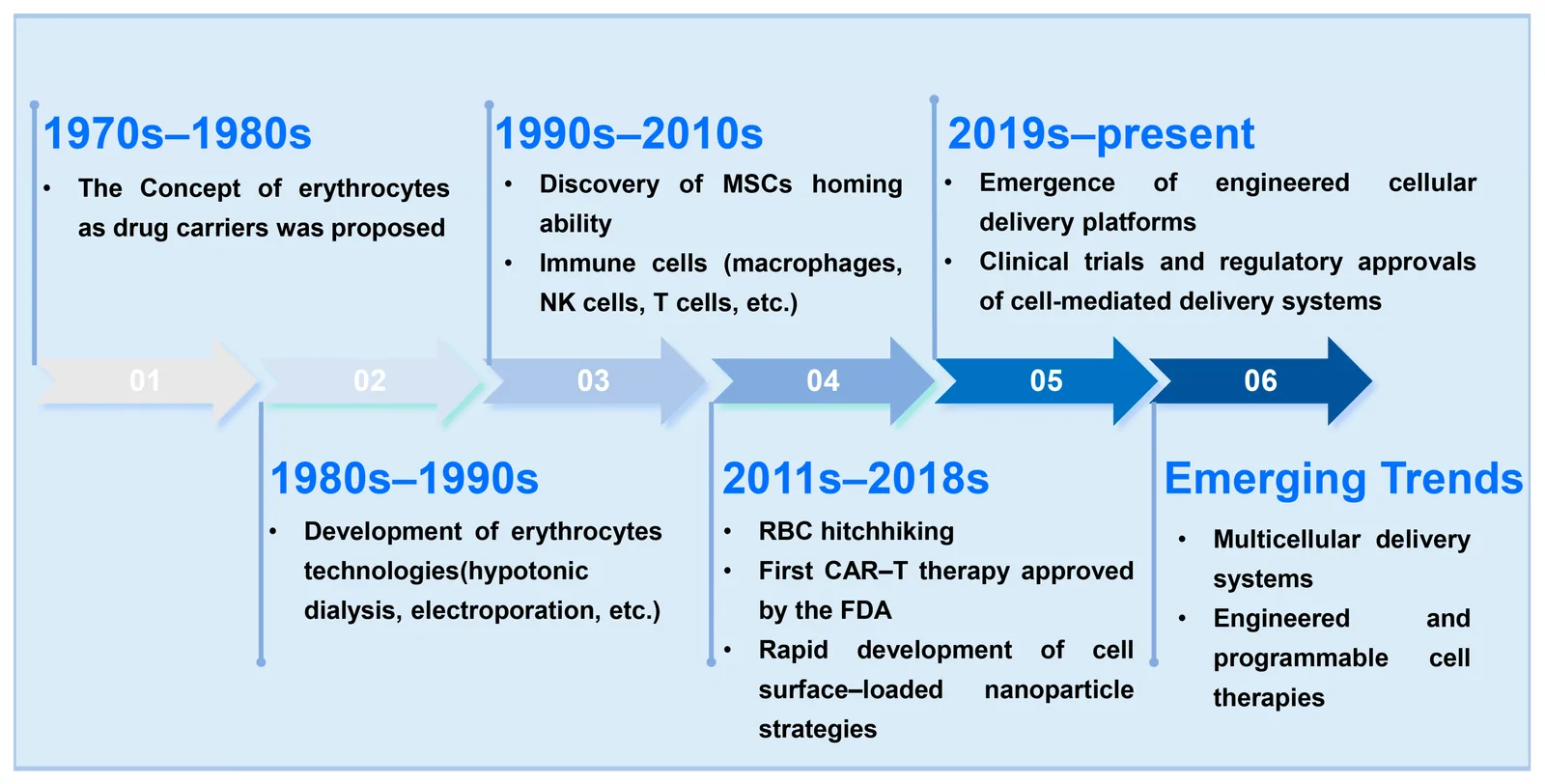
An evolution diagram about cell-mediated drug delivery systems: from erythrocytes to engineered living therapies.

Selecting appropriate cell carriers is crucial for designing effective LCNDDs (Figure 1). Each cell type has unique biological functions and distinct application values in various diseases. Therefore, the commonly used cell carriers in drug delivery systems are summarized below (Table 1).

### 2.1. Erythrocytes

Erythrocytes are the most abundant cells in the blood, with a diameter of approximately 7.5 μm. They exhibit a biconcave disk shape, providing a large surface area for nanoparticle (NPs) loading [41]. In addition, mature erythrocytes lack nuclei and most intracellular organelles, which minimizes the risk of cellular complications. Their large internal volume also enables efficient drug encapsulation. Under high shear forces in the bloodstream, NPs adsorbed on erythrocytes can detach at the first downstream capillary bed. This effect is particularly evident after intravenous administration, where NPs may accumulate in the lungs, making erythrocyte-based delivery suitable for pulmonary diseases [42,43].

**Figure 2:**
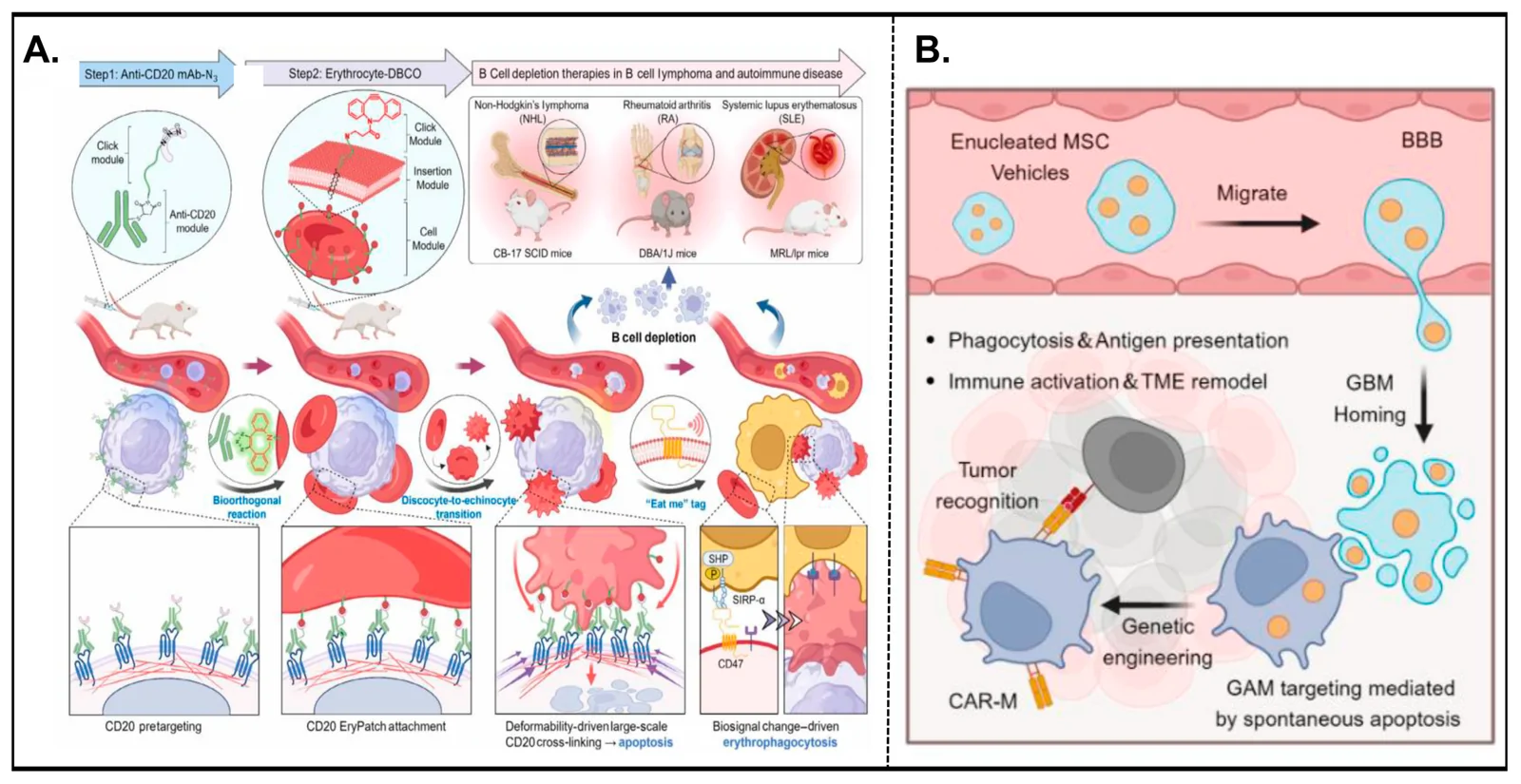
Key biological properties of erythrocytes and MSCs as drug delivery carriers. (**A**) A novel strategy was developed to induce CD20 receptor crosslinking on the cell surface. By exploiting the mechanical stress generated during erythrocyte deformation, micron-scale crosslinking of CD20 receptors was achieved, leading to the targeted elimination of Raji cells [47]. Reprinted with permission from {Liu, J.; Wang, F.; Li, L. Erythrocyte Patch for Enhanced B Cell Depletion Therapy. *Sci. Adv.* **2026**, *12*, eaed3138}. Copyright {2026} American Association for the Advancement of Science (AAAS). (**B**) Enucleated MSCs facilitate the in vivo generation of CAR macrophages in the brain for glioblastoma treatment [84]. Reprinted with permission from {Zhou, L.; Song, Q.; Zhang, X.; Cao, M.; Xue, D.; Sun, Y.; Mao, M.; Li, X.; Zhang, Z.; Liu, J.; et al. In vivo Generation of CAR Macrophages via the Enucleated Mesenchymal Stem Cell Delivery System for Glioblastoma Therapy. *Proc. Natl. Acad. Sci. USA* **2025**, *122*, e2426724122}. Copyright {2025} the National Academy of Sciences.

Normal erythrocytes have a long circulation lifespan and excellent biocompatibility. They can effectively evade clearance by the reticuloendothelial system (RES) and are widely used to improve the pharmacokinetic (PK) and pharmacodynamic (PD) profiles of therapeutic agents [44]. Xie and colleagues developed a thrombolytic targeting system based on artificial polysaccharide-engineered microvesicles. By masking a urokinase-type plasminogen activator (uPA) with erythrocytes, the biosafety and therapeutic efficacy of uPA were significantly enhanced [45]. However, erythrocytes not only prevent rapid drug clearance but also provide a unique opportunity for spleen-targeted delivery. Upon aging or external stress, erythrocytes undergo morphological deformation. Splenic macrophages recognize and clear these altered erythrocytes through reduced CD47-SIRPα signaling, initiating erythrocyte-mediated immune clearance [46]. In an interesting study, Li et al. exploited the mechanical forces generated by erythrocyte deformation to induce extensive CD20 receptor clustering on human Burkitt lymphoma cells (Raji cells), thereby triggering apoptosis (Figure 2). Meanwhile, downregulation of CD47 on deformed erythrocytes further promoted macrophage-mediated clearance. Therefore, drug-loaded deformed erythrocytes may serve as a promising platform for spleen-targeted therapy and vaccine delivery [47].

Based on these biological characteristics, erythrocyte-based delivery systems have shown considerable potential in a range of therapeutic applications, including long-acting drug delivery for chronic diseases, treatment of hematological malignancies, enzyme replacement therapy for metabolic disorders and vaccine delivery that exploits uptake by MPS. Despite these advantages, several challenges remain, including limited drug-loading capacity, compromised structural stability, immunological safety concerns and difficulties in achieving controlled cargo release. For example, erythrocytes lack intrinsic migratory and chemotactic capabilities, which restricts their ability to penetrate deeply into solid tumor tissues [48]. Furthermore, inappropriate drug-loading procedures may damage erythrocyte membranes and alter their physiological properties, resulting in premature MPS-mediated clearance of the carrier cells and consequently reduced delivery efficiency and therapeutic efficacy.

### 2.2. Platelets

Platelets are anucleate cell fragments derived from bone marrow megakaryocytes, with a circulating lifespan of approximately 7–10 days [49]. They exhibit excellent biocompatibility, prolonged circulation and disease-targeting capabilities. Platelets play essential roles in hemostasis, vascular repair, tumor progression and immune regulation [50,51].

The surface of platelets is enriched with various functional receptors and membrane proteins, including integrins (α_IIb_β_3_ and α_2_β_1_), selectins (P-selectin) and C-type lectin-like receptor 2 (CLEC-2) [52]. Platelets possess intrinsic pathological homing properties and can be activated upon sensing local vascular injury or inflammatory microenvironments. After activation, platelet surface P-selectin interacts with ligands such as P-selectin glycoprotein ligand-1 (PSGL-1) and CD44 on tumor cells. This interaction promotes platelet accumulation around tumor tissues and circulating tumor cells (CTCs) [53,54,55,56,57]. Based on their tumor-homing ability, platelet-based delivery systems are commonly developed for the delivery of immune checkpoint inhibitors. In addition, platelets exhibit excellent immune evasion properties. High expression of CD47 on platelet surfaces allows them to escape clearance by MPS through the CD47–SIRPα axis, thereby prolonging their circulation time. Beyond these functions, platelets also show promising applications in wound healing, bone regeneration and myocardial repair [58]. Platelets contain abundant growth factors, including vascular endothelial growth factor (VEGF), platelet-derived growth factor (PDGF) and transforming growth factor-β (TGF-β). These factors promote angiogenesis, cell proliferation and extracellular matrix remodeling. They also regulate inflammatory microenvironments and stem cell recruitment. Wang et al. developed a macrophage–platelet hybrid delivery system. In this system, platelets transferred functional mitochondria to macrophages at injury sites, restoring macrophage energy metabolism and efferocytosis. This strategy significantly reduced myelin debris accumulation and promoted axonal remyelination [59].

Because of their unique biological functions, platelets have shown great potential as drug carriers for tumor-targeted delivery, prevention of postoperative cancer metastasis, antithrombotic therapy and wound repair. However, several limitations remain. Platelets are prone to nonspecific activation upon temperature changes and chemical modification. This might cause premature drug release. In addition, although platelets have a high affinity for circulating tumor cells (CTCs), their role in cancer therapy is bidirectional. They can suppress tumor progression but also promote tumor growth and metastasis via multiple mechanisms. Moreover, transfusion of large numbers of platelets also increases the risk of thrombosis.

### 2.3. Macrophages

Macrophages are essential innate immune cells that are widely distributed in tissues, including the spleen, liver and lungs. They play critical roles in pathogen clearance, tissue repair and immune regulation [60]. In recent years, macrophages have emerged as promising living cell carriers for drug delivery [61,62]. This is attributed to their intrinsic disease-homing ability, capacity for trans-barrier migration, potent phagocytic activity and immunomodulatory functions.

Macrophages exhibit strong inflammatory and tumor-homing abilities. Within the tumor microenvironment (TME), tumor cells and stromal cells continuously secrete chemokines, including CCL2, CCL5, CCL7 and CXCL12. These chemokines interact with receptors such as CCR2, CCR5 and CXCR4 on macrophages, thereby promoting macrophage recruitment and infiltration into tumor sites [63,64]. In addition, solid tumors often contain extensive hypoxic regions. Hypoxia-inducible factor-1α (HIF-1α) upregulates the expression of chemotactic signals, including VEGF, CXCL12 and CSF-1, further enhancing macrophage migration into deep tumor regions [65]. Unlike conventional nanomedicines that mainly rely on passive diffusion, macrophages can actively cross vascular barriers and dense tumor extracellular matrices. This ability enables deep penetration into tumor cores and delivery of nanomedicines to regions that are difficult to access by traditional nanocarriers [66]. Macrophages exhibit high phenotypic plasticity and can dynamically switch between pro-inflammatory M1-like and anti-inflammatory M2-like states in response to environmental signals. Tumor-associated macrophages (TAMs) predominantly exhibit an M2-like phenotype, which promotes angiogenesis, tumor invasion and immune suppression. Therefore, reprogramming TAMs toward an M1-like phenotype by delivering immune-stimulatory agents, such as interferon-γ (IFN-γ), has become an important strategy for enhancing antitumor immunity [67]. The Samir Mitragotri group developed a macrophage surface-attached “backpack” delivery system. This system continuously released IFN-γ to maintain macrophage polarization toward an M1-like phenotype, thereby improving antitumor immune responses [68]. Chen et al. developed a macrophage-based delivery system carrying GeS nanosheets. The sonosensitizer-loaded nanomaterials were conjugated to macrophage surfaces through maleimide–thiol chemistry. The tumor-homing ability of macrophages enabled targeted accumulation in tumor tissues. Upon ultrasound stimulation, the system induced tumor cell apoptosis and remodeled the tumor microenvironment [69].

Because of these biological properties, macrophages have been explored as delivery platforms for cancer therapy, autoimmune diseases and neurological disorders. Despite their unique advantages, several challenges limit their clinical translation. Firstly, different macrophage phenotypes have different biological functions. Within the TME, therapeutic macrophage carriers might be reprogrammed into an M2/TAM-like state, which can reduce or even counteract therapeutic effects. Moreover, nanocarriers loaded on the macrophage surface or internalized within macrophages might undergo degradation after phagocytosis. This issue is particularly important for biomacromolecules, including mRNA, siRNA and protein therapeutics, as their biological activities might be compromised during intracellular processing [70].

### 2.4. Immune Cells

#### 2.4.1. T Lymphocytes

T cells are key components of the adaptive immune system. Their T cell receptors (TCRs) recognize tumor antigens by binding to peptide–MHC complexes presented on target cells. Upon activation, T cells eliminate target cells through the release of perforin, granzymes and interferon-γ (IFN-γ) [71]. T cells express various functional membrane proteins, including CD3, CD28, 4-1BB, OX40 and PD-1. These molecules regulate T cell activation, proliferation and immune responses. They also provide anchoring sites for engineering nanocarrier attachment [72]. T cells can respond to chemokine signals in tumor and inflammatory microenvironments. This enables active migration and accumulation at disease sites, making them promising living cell carriers for drug delivery. Darrell J. Irvine and colleagues exploited CCR7-mediated lymph node homing of T cells. They loaded SN-38-containing nanogels onto T cell surfaces, which significantly enhanced SN-38 accumulation and retention in lymphoma tissues [73]. Beyond cancer therapy, T cells have also attracted increasing attention in tissue repair and regeneration. Regulatory T cells (Tregs) can promote tissue regeneration by regulating the balance of monocytes and macrophages in injured tissues [74]. T cells can cross BBB under both physiological and pathological conditions. This property provides a potential strategy for delivering small molecules and nanomedicines that are limited by the CNS barriers. Wendell A. Lim and colleagues developed a tissue-responsive T cell platform for brain disease therapy. By engineering synthetic Notch receptors on T cell surfaces, this system enabled selective activation of therapeutic gene expression in response to tissue signals [75].

Although T cells play critical roles in cancer immunotherapy, their application in solid tumors remains challenging. Tumor cells employ multiple mechanisms to evade immune surveillance, leading to insufficient T cell infiltration, immunosuppression and T cell exhaustion. Currently, adoptive T cell therapies (ACT), including tumor-infiltrating lymphocyte (TIL) therapy, TCR-T, CAR-T and next-generation engineered ACT approaches, are under extensive investigation and show potential for clinical translation.

#### 2.4.2. Natural Killer (NK) Cells

NK cells can directly recognize and eliminate abnormal cells without prior antigen sensitization or major histocompatibility complex (MHC) restriction. Activating receptors, such as CD16, NKG2D, NKp30, NKp44 and NKp46, contribute to tumor recognition and cytotoxic responses, while inhibitory receptors, including KIRs, NKG2A and TIGIT, maintain immune homeostasis by limiting excessive NK cell activation [76,77]. These receptors are involved in NK cell activation and tumor recognition. They also provide important targets for antibody-based therapies, nanomedicine engineering and surface modification. Recently, researchers have developed bispecific and multispecific antibodies that simultaneously bind NK cell receptors and tumor-associated antigens. These strategies promote NK cell–tumor cell interactions and enhance antitumor responses. Gauthier et al. developed a trifunctional NK cell engager (NKCE) targeting NKp46, CD16 and tumor antigens. This platform enhanced NK cell recruitment and activation, resulting in improved NK cell-mediated tumor killing. It also provided a foundation for NK cell engager-based therapies [78]. Several bispecific and multispecific antibodies derived from NKCE strategies have entered clinical trials. They have shown promising therapeutic potential in hematological malignancies, including relapsed or refractory lymphomas [79]. In recent years, chimeric antigen receptor-engineered NK cells (CAR-NK) have attracted increasing attention due to their low toxicity, lack of human leukocyte antigen (HLA) matching requirements and potent antitumor activity [80]. Zhao et al. developed a nanoengineered CAR-NK cell platform. Near-infrared-II (NIR-II) nanomedicines were conjugated to CAR-NK cell surfaces as “backpacks” through click chemistry. This strategy enabled precise tumor navigation and enhanced immunotherapy for lung cancer [81].

Currently, NK cell-based therapies mainly focus on cancer treatment. Compared with T cells, NK cells recognize tumors in an MHC-independent manner and show a lower risk of cytokine release syndrome (CRS). However, NK cells face similar challenges to T cells in solid tumors, including limited tumor infiltration and immunosuppressive tumor microenvironments. In addition, NK cells have shorter lifespans and lower gene-editing efficiency. Combining NK cells with advanced engineering technologies may further enhance their antitumor activity and clinical translation potential.

### 2.5. Mesenchymal Stem Cells (MSCs)

MSCs are stem cells with multipotent differentiation capacity and immunomodulatory functions. They are widely distributed in tissues, including bone marrow, adipose tissue, umbilical cord and placenta [82]. In addition to their differentiation potential, MSCs exhibit strong paracrine activity. They secrete cytokines, growth factors, chemokines and adhesion molecules to promote tissue regeneration, angiogenesis and immune regulation. These effects contribute to the improvement of the tissue repair microenvironment [83]. MSCs also exhibit intrinsic tropism toward tumor and inflammatory sites. Shi et al. utilized enucleated MSCs to deliver CAR-encoding plasmids to glioma tissues. This strategy enabled in vivo reprogramming of glioma-associated microglia/macrophages (GAMs) through targeted delivery [84]. MSCs express various chemokine receptors, among which the CXCR4/SDF-1 axis plays a key role in their migration toward injured tissues and tumor microenvironments [85,86]. This process involves multiple steps, including selectin-mediated rolling, integrin-mediated adhesion, transendothelial migration and directional movement through the extracellular matrix [87]. Under hypoxic conditions, hypoxia-inducible factor-1α (HIF-1α) upregulates SDF-1 expression, thereby promoting MSC infiltration into deep hypoxic tumor regions and penetration into solid tumor cores [88]. MSCs also exhibit low immunogenicity and strong immunoregulatory properties. They express low levels of major histocompatibility complex class II (MHC II) molecules and costimulatory molecules, including CD40, CD80 and CD86. Meanwhile, they retain basal expression of MHC class I molecules. These characteristics allow MSCs to partially evade host immune recognition and clearance, making them suitable for allogeneic transplantation and cell-based therapies [89].

Recently, MSCs have been widely explored as targeted delivery vehicles for anticancer therapeutics. Their intrinsic regenerative functions also support applications in tissue engineering and regenerative medicine [90]. In addition, MSC-mediated immunomodulation has been investigated for the treatment of autoimmune diseases, including graft-versus-host disease (GVHD), Crohn’s disease and multiple sclerosis. However, MSCs may also promote tumor progression under certain conditions. Moreover, their fate after administration remains difficult to control. Therefore, the therapeutic application of MSCs requires careful consideration of both their advantages and potential risks.

### 2.6. Bacteria

Bacterial therapy has attracted increasing attention in cancer treatment due to its unique advantages compared with conventional nanocarriers and mammalian cell-based delivery systems [91]. Unlike other living cell carriers, bacteria possess an intrinsic tumor-targeting ability, active motility, immune-stimulatory activity and genetic programmability [92].

The most prominent advantage of bacteria is their natural colonization ability in hypoxic tumor microenvironments. Facultative anaerobic bacteria, such as attenuated Salmonella typhimurium VNP20009 and obligate anaerobic bacteria, such as Bifidobacterium, can be rapidly cleared from normal tissues after systemic or oral administration. However, they preferentially accumulate and proliferate within immunosuppressive, hypoxic and necrotic regions of solid tumors. This results in significantly higher bacterial colonization levels in tumors than in normal tissues [93]. Therefore, compared with other cell-based carriers that rely mainly on receptor recognition or inflammatory chemotaxis, bacteria exhibit stronger penetration and accumulation in deep tumor regions. Some bacteria also possess self-propelled motility driven by flagella. Unlike nanocarriers that mainly depend on blood circulation and passive diffusion, motile bacteria can actively migrate along chemical gradients. They can penetrate dense extracellular matrices and overcome high interstitial pressure barriers, thereby improving intratumoral distribution and penetration depth [94]. Bacteria also exhibit intrinsic immunostimulatory activity. Bacterial components, including lipopolysaccharides (LPS), flagellin, CpG DNA and peptidoglycan, are recognized by Toll-like receptors (TLRs) on host cells and cytosolic NOD-like receptors (NLRs). This activates NF-κB and IRF signaling pathways, promotes dendritic cell maturation, enhances antigen cross-presentation and induces the production of type I interferons and pro-inflammatory cytokines [95,96]. Therefore, bacteria can function as both drug carriers and natural immune adjuvants. They can convert immunosuppressive “cold tumors” into immune-active “hot tumors”. This immune activation can further enhance therapeutic efficacy when combined with immune checkpoint inhibitors. In addition, bacteria provide a highly programmable genetic platform. Genetic engineering enables the integration of therapeutic gene modules into bacterial genomes. Engineered bacteria can locally produce and release therapeutic proteins, such as TNF-α, IL-2 and TRAIL, prodrug-converting enzymes, such as cytosine deaminase, or RNA interference molecules within tumors. This transforms bacteria from simple drug carriers into “miniature drug factories” [97].

Given these unique biological properties, bacteria provide new opportunities for cancer therapy. However, their application as drug delivery vehicles is limited by safety concerns. Systemic administration of live bacteria may cause infection, toxicity and uncontrolled bacterial proliferation. To improve biosafety, engineering strategies have introduced multiple control mechanisms, including auxotrophic systems (e.g., purine deficiency and D-amino acid dependency), temperature-sensitive replication systems and inducible suicide switches (e.g., arabinose-inducible bacterial lysis systems). These approaches enable better control over bacterial proliferation and clearance in vivo [98]. Nevertheless, the fate and long-term behavior of engineered bacteria after administration require further investigation.

## 3. Construction Strategies of LCNDDs

**Figure 3:**
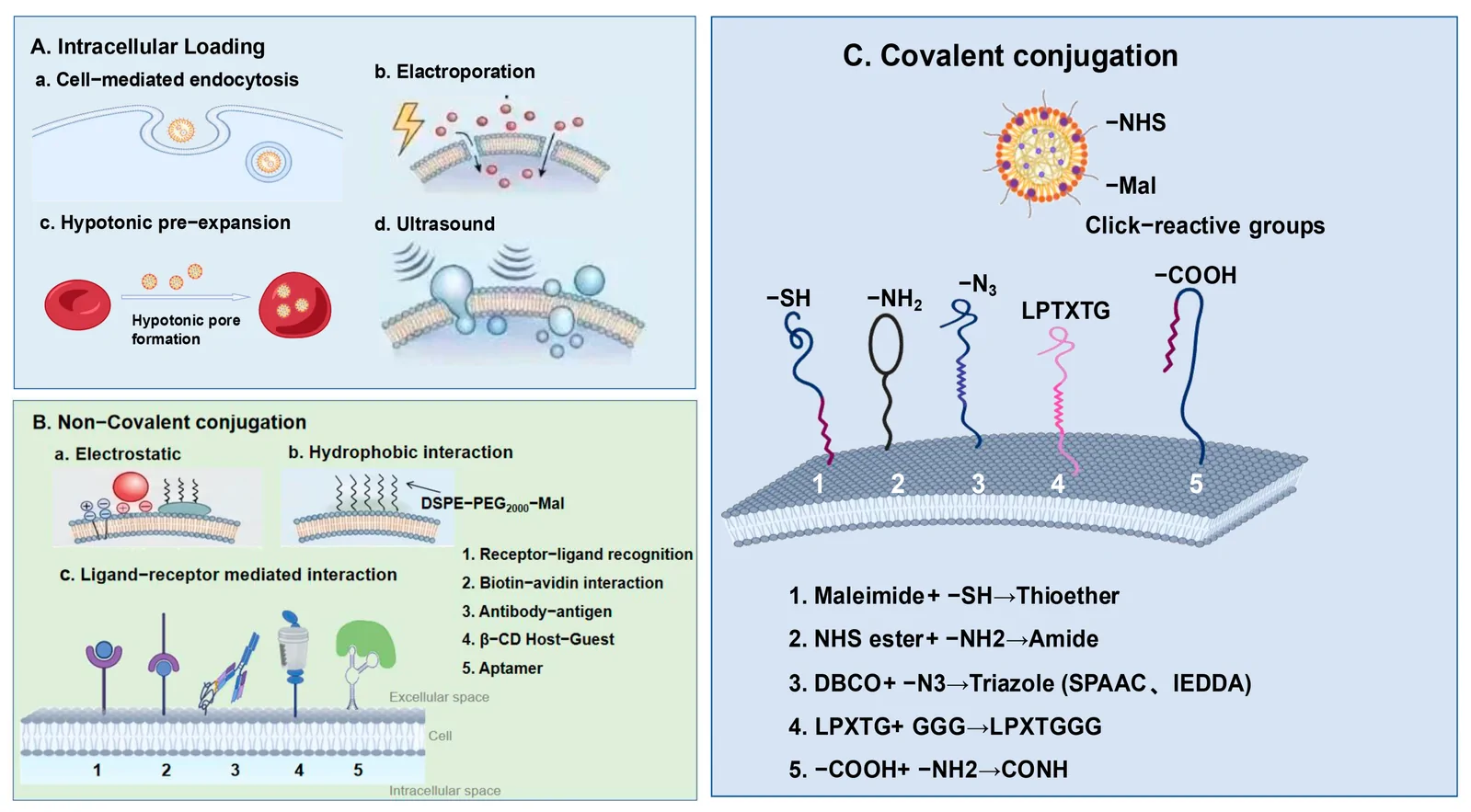
Major strategies for cell–nanodrug conjugation.

The design of hitchhiking systems provides new opportunities for the treatment of challenging diseases. In these systems, living cells and nanocarriers serve as two independent modules and work synergistically to combine their respective advantages. The main construction strategies include intracellular loading, non-covalent modification and covalent conjugation (Figure 3). Selecting appropriate cell carriers and coupling strategies is critical for achieving selective drug accumulation at target sites and improving therapeutic efficacy. In addition, responsive modification strategies that enhance cytosolic delivery have also been discussed.

### 3.1. Intracellular Loading

**Figure 4:**
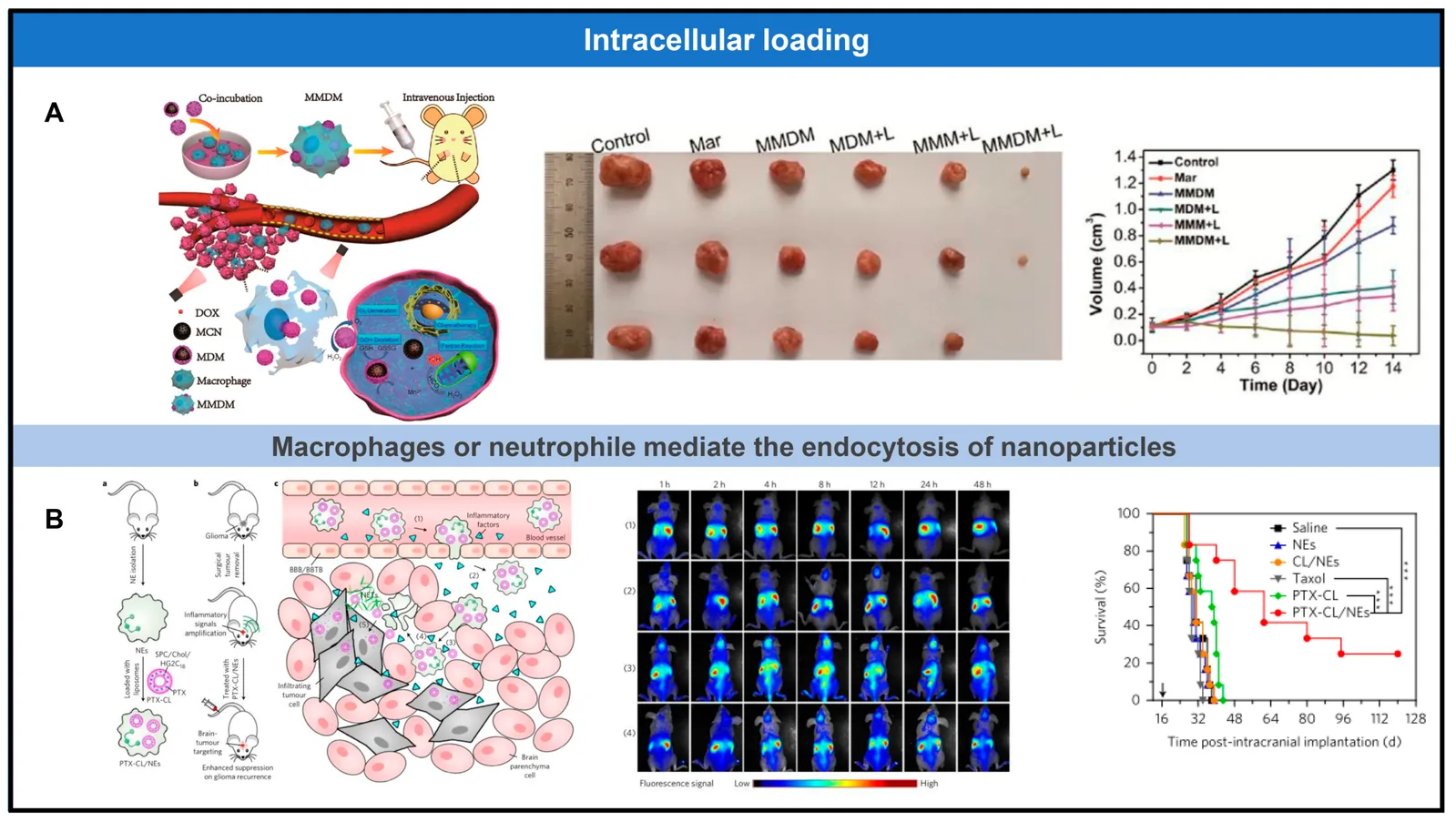
Common strategies for intracellular loading of NCs. (**A**) A self-destructive macrophage carrier that migrates to the tumor after internalizing nanoparticles, enabling synergistic chemo-phototherapy [104]. Reprinted with permission from {Sun, P.; Deng, Q.; Kang, L.; Sun, Y.; Ren, J.; Qu, X. A Smart Nanoparticle-Laden and Remote-Controlled Self-Destructive Macrophage for Enhanced Chemo/Chemodynamic Synergistic Therapy. *ACS Nano* **2020**, *14*, 13894–13904}. Copyright {2020} American Chemical Society (ACS). (**B**) A neutrophil carrier loaded with paclitaxel (PTX)-containing liposomes penetrates the brain and suppresses glioma recurrence after surgical resection [105]. The a refers to the Schematic illustration of the preparation of PTX-containing formulations. The b refers to the Schematic that shows how the PTX-containing formulations suppress postoperative glioma recurrence in mice. The c refers to the Schematic that shows how PTX-containing formulations target glioma after intravenous injection into mice whose brain tumour has been resected surgically. *** refers to *p* < 0.001 and the arrow in survival curve indicates the time of the surgery. Reprinted with permission from {Xue, J.; Zhao, Z.; Zhang, L.; Xue, L.; Shen, S.; Wen, Y.; Wei, Z.; Wang, L.; Kong, L.; Sun, H.; et al. Neutrophil-Mediated Anticancer Drug Delivery for Suppression of Postoperative Malignant Glioma Recurrence. *Nat. Nanotechnol.* **2017**, *12*, 692–700}. Copyright {2017} Springer Nature.

Intracellular loading provides a strategy for directly incorporating nanomedicines into living cells (Figure 4). This approach can reduce drug clearance and nonspecific interactions with tissues while improving drug stability and in vivo retention. However, it may also affect cell viability [99]. Intracellular loading is mainly achieved through two approaches. The first approach relies on the endocytic or phagocytic capacity of immune cells to internalize NCs [100]. The second approach uses physical stimuli to transiently increase cell membrane permeability, including electroporation, ultrasound and mechanical extrusion [101,102]. Erythrocytes are highly sensitive to osmotic changes. Therefore, NCs can be loaded into erythrocytes through hypotonic pre-swelling or hypotonic dialysis methods [103].

#### 3.1.1. Immune Cell-Mediated Endocytosis/Phagocytosis

The physicochemical properties of NCs, including particle size, surface charge and hydrophilicity/hydrophobicity, can significantly affect cellular uptake efficiency [106]. Macrophage uptake of nanomedicines mainly relies on phagocytosis and multiple endocytic pathways, including clathrin-mediated endocytosis (CME), fast endophilin-mediated endocytosis (FEME) and clathrin-independent carrier/GPI-anchored protein-enriched early endosomal compartment (CLIC/GEEC) pathways. Generally, macrophages prefer phagocytose nanoparticles with sizes larger than 500 nm, while the particle size should remain below 10 μm [107]. In contrast, nanoparticles smaller than 200 nm are mainly internalized through endocytic pathways [108]. Chen et al. synthesized a biodegradable photoluminescent polyester (BPLP-PLA) polymer and loaded it with the BRAF V600E mutation-specific inhibitor PLX4032. They further modified the nanoparticles with macrophage-targeting peptide (MTP) to enhance nanoparticle uptake by THP-1 cells. Approximately 96% of THP-1 cells successfully internalized MTP-modified BPLP-PLA-PLX4032 nanoparticles. The drug-loaded macrophages delivered nanoparticles to melanoma sites through interactions with tumor cells. This strategy reduced tumor cell viability to approximately 30% and showed potent antitumor activity [109]. However, intracellular loading through immune cell-mediated endocytosis or phagocytosis also has several limitations. First, internalized nanoparticles are transported to the endosomal–lysosomal pathway. The acidic environment and hydrolytic enzymes may cause carrier degradation or premature drug release, reducing drug availability at disease sites. Second, the intracellular drug loading capacity is limited by the phagocytic capacity of macrophages. Excessive nanoparticle uptake may impair cell viability and chemotactic migration, thereby reducing their homing ability for diseased tissues [110].

#### 3.1.2. Erythrocyte Intracellular Loading

Erythrocytes can sense changes in extracellular osmotic pressure and undergo reversible shrinkage or swelling under hypertonic or hypotonic conditions [111]. When exposed to a hypotonic environment, transient nanoscale pores (approximately 20–200 nm) are formed on the erythrocyte membrane. These pores allow the entry of small molecules, proteins and small-sized NPs into erythrocytes [112,113]. Subsequent isotonic restoration closes the membrane pores and enables drug encapsulation [114]. Hamidi et al. prepared chitosan nanogels using an ionotropic gelation method to encapsulate the antiepileptic drug sodium valproate. The drug-loaded nanogels were then introduced into intact erythrocytes through hypotonic dialysis, generating a “nano–cell” hybrid delivery system. This system prolonged drug activity and showed potential for sustained intravenous delivery of sodium valproate for long-term epilepsy treatment [115]. Although erythrocytes have a simple structure and a large intracellular volume, their lack of endocytic capacity limits intracellular drug loading. The entry of therapeutic agents is mainly controlled by membrane transport mechanisms, which restricts the clinical application of erythrocyte-based intracellular loading strategies. In addition, erythrocytes are sensitive to external stimuli and may undergo irreversible damage, including hemolysis, deformation and loss of membrane integrity. Therefore, a balance between drug loading capacity and erythrocyte viability must be carefully optimized.

### 3.2. Non-Covalent Conjugation

**Figure 5:**
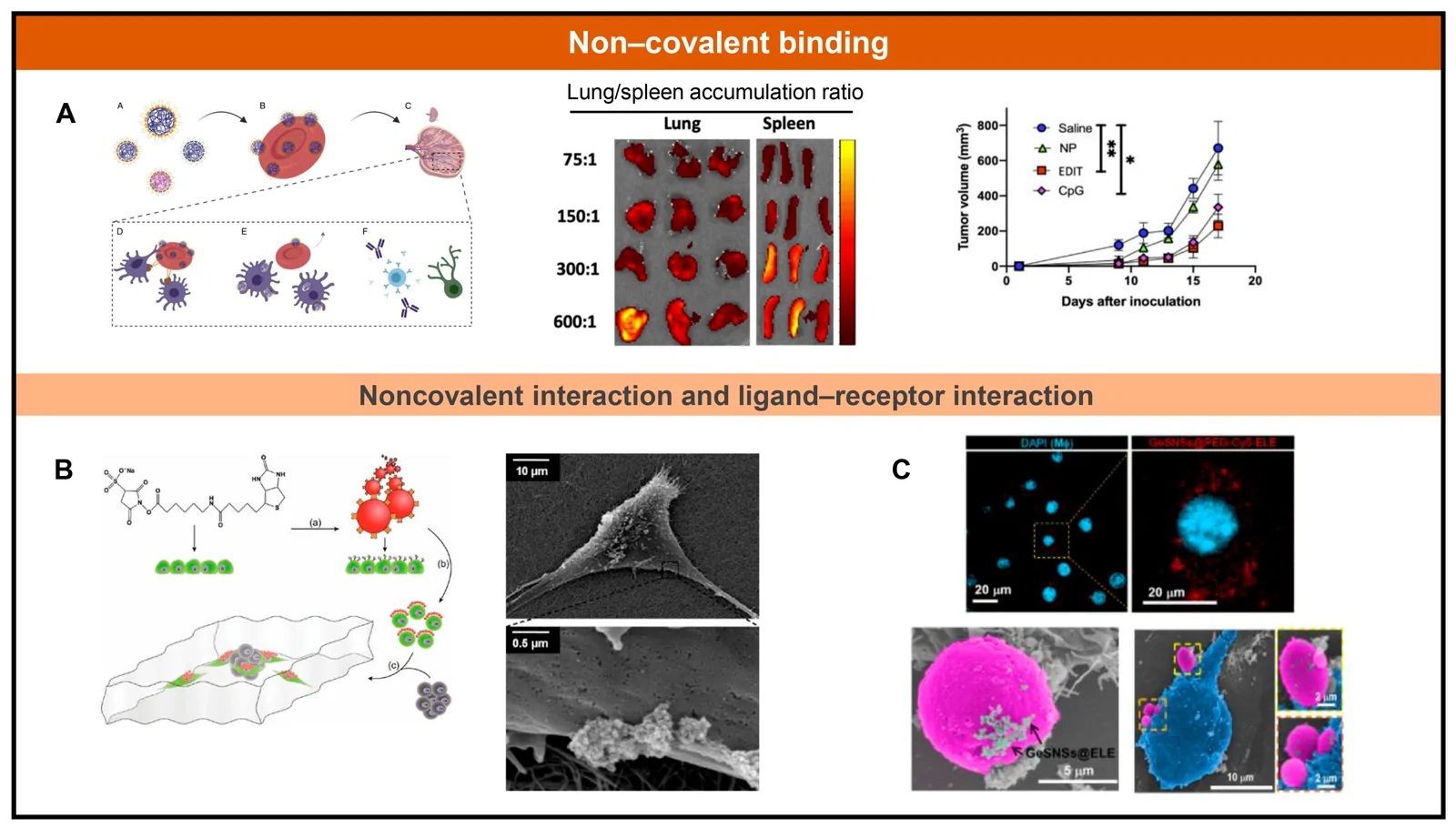
Common strategies for non-covalent binding of NCs. (**A**) An erythrocyte-driven immune targeting (EDIT) strategy enables the presentation of nanoparticle cargos to splenic APCs. * and ** refer to *p* < 0.05 and *p* < 0.01, respectively [116]. Reprinted with permission from {Ukidve, A.; Zhao, Z.; Fehnel, A.; Krishnan, V.; Pan, D.C.; Gao, Y.; Mandal, A.; Muzykantov, V.; Mitragotri, S. Erythrocyte-Driven Immunization via Biomimicry of Their Natural Antigen-Presenting Function. *Proc. Natl. Acad. Sci. USA* **2020**, *117*, 17727–17736.} Copyright {2020} The National Academy of Sciences. (**B**) A technique to create nanoparticulate cellular patches that remain attached to the membrane of cells for up to 2 days [117]. Reprinted with permission from {Cheng, H.; Kastrup, C.J.; Ramanathan, R.; Siegwart, D.J.; Ma, M.; Bogatyrev, S.R.; Xu, Q.; Whitehead, K.A.; Langer, R.; Anderson, D.G. Nanoparticulate Cellular Patches for Cell-Mediated Tumoritropic Delivery. *ACS Nano* **2010**, *4*, 625–631.} Copyright {2010} American Chemical Society (ACS). (**C**) A macrophage hitchhiking strategy combines chemotherapy and sonodynamic therapy to synergistically kill tumor cells and remodel the tumor microenvironment [69]. Reprinted with permission from {Chen, S.; Li, Y.; Zhou, Z.; Saiding, Q.; Zhang, Y.; An, S.; Khan, M.M.; Ji, X.; Qiao, R.; Tao, W.; et al. Macrophage Hitchhiking Nanomedicine for Enhanced β-Elemene Delivery and Tumor Therapy. *Sci. Adv.* **2025**, *11*, eadw7191.} Copyright {2025} American Association for the Advancement of Science (AAAS).

Non-covalent conjugation utilizes intermolecular non-covalent interactions to anchor NCs onto the surface of living cells (Figure 5). This approach offers significant advantages, including mild conditions, no requirement for chemical modification of membrane proteins and preservation of cell viability, making it one of the important strategies for constructing cell–nanocarrier conjugates. Currently employed non-covalent conjugation strategies primarily include nonspecific adsorption, lipid insertion, receptor–ligand binding, antigen–antibody binding and biotin–avidin interactions.

#### 3.2.1. Nonspecific Adsorption

Nanocarrier adsorption onto cell surfaces is a physical process driven by spontaneous interactions between NCs and cell membranes. This process mainly depends on weak intermolecular forces, including electrostatic interactions, hydrophobic interactions and van der Waals forces. Cell membranes are primarily composed of phospholipid bilayers and contain negatively charged phosphatidylserine (PS) and phosphate groups [118]. In addition, sialic acid residues in the glycocalyx contribute to the negative surface charge of cell membranes. Positively charged nanoparticles can interact with negatively charged glycocalyx through electrostatic attraction, leading to strong adsorption onto cell surfaces. Cell membranes also contain various transmembrane proteins and glycoproteins. These molecules create alternating hydrophilic and hydrophobic regions on the membrane surface. Local hydrophobic domains provide binding sites for hydrophobic interactions [119]. Hydrophobic NCs tend to insert into or embed within the hydrophobic core of the cell membrane and achieve stable attachment through hydrophobic interactions. Zhao et al. developed an erythrocyte-leveraged chemotherapy (ELeCt) platform for the treatment of early and advanced lung metastasis. In this system, doxorubicin-loaded NCs were adsorbed onto erythrocyte surfaces. The adsorption was attributed to nanoparticle-induced membrane deformation, which generated surface tension and promoted non-covalent interactions between nanoparticles and erythrocytes. Taking advantage of the long circulation ability of erythrocytes and the high shear forces within narrow pulmonary capillaries, nanoparticles were released from erythrocytes in a shear-responsive manner. This strategy effectively inhibited both early and advanced melanoma lung metastasis and prolonged the survival of tumor-bearing mice [120]. Furthermore, studies have shown that biocompatible ionic liquid coatings, such as choline trans-2-hexenoate (C2HA), can enhance nanoparticle adsorption onto erythrocytes [121].

#### 3.2.2. Ligand–Receptor and Biomolecular Pair-Mediated Conjugation

This type of hitchhiking system connects nanocarriers to cell surfaces through ligand–receptor interactions. Ligand–receptor binding usually exhibits high affinity and can reduce nanocarrier detachment caused by blood shear forces. Hyaluronic acid (HA), a natural ligand of the CD44 receptor, can anchor nanocarriers onto various immune cells through HA–CD44 interactions. In addition, HA–CD44 signaling can regulate cellular functions [122]. Hlaing et al. designed HA-functionalized nanoparticles co-loaded with curcumin. These nanoparticles interacted with CD44-expressing intestinal epithelial cells and accumulated in inflamed colitis tissues. They subsequently targeted activated macrophages for ulcerative colitis treatment. HA modification enhanced nanoparticle targeting to intestinal macrophages and significantly increased the cellular uptake [123]. However, receptor expression on cell surfaces is dynamic and can be influenced by inflammation, cellular states and infections. This variability may reduce ligand–receptor binding stability and drug loading efficiency [124].

Antigen–antibody recognition relies on the specific binding of antibody Fab regions to cell surface antigens. This strategy enables the specific anchoring of nanocarriers onto target cells. Darrell J. Irvine et al. used anti-CD45 antibodies to attach nanogels loaded with human interleukin (IL)-15 superagonist (IL-15Sa) onto T cell surfaces. This system enabled T cell receptor (TCR)-responsive drug release. The “on-demand delivery” strategy allowed localized drug release at activated T cell sites, thereby reducing systemic exposure and associated toxicities [125]. Antibody–antigen recognition provides high targeting specificity. Antibody binding can also trigger receptor-mediated endocytosis for certain surface receptors. Therefore, this strategy enables not only cell surface attachment but also intracellular delivery of therapeutic cargos [126]. It is particularly valuable for biomacromolecules, including nucleic acids, proteins and peptides, which require intracellular activity.

The biotin–(strept)avidin system is one of the strongest non-covalent interactions, with an extremely high binding affinity (K_d_ ≈ 10^−15^ M). Its binding strength exceeds that of most antigen–antibody interactions. This high affinity results from the precise insertion of biotin into the hydrophobic binding pocket of avidin or streptavidin. The interaction is further stabilized by hydrogen bonding and van der Waals forces and shows limited sensitivity to pH, temperature and organic solvents [127]. In cell–nanocarrier conjugation, this system is commonly applied as a modular bridging strategy. Nanocarriers are first biotinylated, while biotin or avidin molecules are introduced onto cell surfaces through lipid insertion or enzymatic modification. Streptavidin or avidin then serves as a bridge to connect cells and nanocarriers [128]. Jacob M. Berlin et al. developed a neural stem cell (NSC)-based delivery system carrying pH-responsive nanoparticles. They oxidized sialic acids on NSC surfaces and introduced biotin groups. The nanoparticles contained biotin-PEG-PDPAEMA, enabling the connection between biotinylated NSCs and nanoparticles through avidin bridging [129]. However, the biotin–streptavidin system may induce immune responses. Avidin derived from non-egg sources, which exhibits lower nonspecific binding, may be more suitable for drug delivery applications [130].

Aptamers are short single-stranded DNA or RNA molecules selected by in vitro systematic evolution of ligands by exponential enrichment (SELEX). They form unique secondary structures, such as G-quadruplexes and hairpins, and bind target membrane proteins with high affinity (K_d_ at the nM to pM range) and specificity [131]. Aptamers can be directly conjugated to nanocarrier surfaces through covalent modification. Alternatively, hydrophobic groups such as cholesterol can be attached to aptamer termini, allowing aptamer–nanocarrier complexes to anchor onto cell membranes through lipid insertion. AS1411 is one of the most widely studied DNA aptamers. It forms a G-quadruplex structure and specifically recognizes nucleolin, which is overexpressed on tumor cell surfaces. AS1411-functionalized liposomes, PLGA nanoparticles, mesoporous silica nanoparticles and gold nanoparticles have been developed for targeted delivery of chemotherapeutic agents, including doxorubicin (DOX), paclitaxel (PTX) and gemcitabine (GEM) [132]. Aptamers can also be displayed multivalently through DNA nanostructure assembly. Qi et al. developed cyclic aptamer assemblies for selective recognition of EpCAM-positive cells. By regulating aptamer valency, this system distinguished cells with different target expression levels [133]. However, in vivo applications of aptamers face challenges such as nuclease degradation and competitive binding by plasma proteins. Chemical modifications, including 2′-fluoro and 2′-O-methyl modifications, as well as PEGylation, are commonly used to improve stability and reduce interference [134].

β-Cyclodextrin (β-CD) contains a hydrophilic outer surface and a hydrophobic cavity. It can act as a host molecule to encapsulate various hydrophobic guest molecules, such as ferrocene (Fc) [135]. The β-CD/Fc system exhibits redox responsiveness. Reduced Fc binds strongly to β-CD, whereas oxidized Fc^+^ shows weaker binding due to increased positive charge and reduced hydrophobicity. Therefore, the β-CD/Fc system can be used to construct ROS-responsive delivery platforms. Wang et al. reported an inflammation-responsive supramolecular erythrocyte hitchhiking system. In this system, β-CD-modified erythrocytes and Fc-modified liposomes were connected through β-CD/Fc host–guest interactions. This system significantly prolonged the circulation half-life of liposomes. Meanwhile, high ROS levels in inflamed lungs triggered responsive separation of liposomes from erythrocytes, enabling site-specific release [136].

### 3.3. Covalent Conjugation

**Figure 6:**
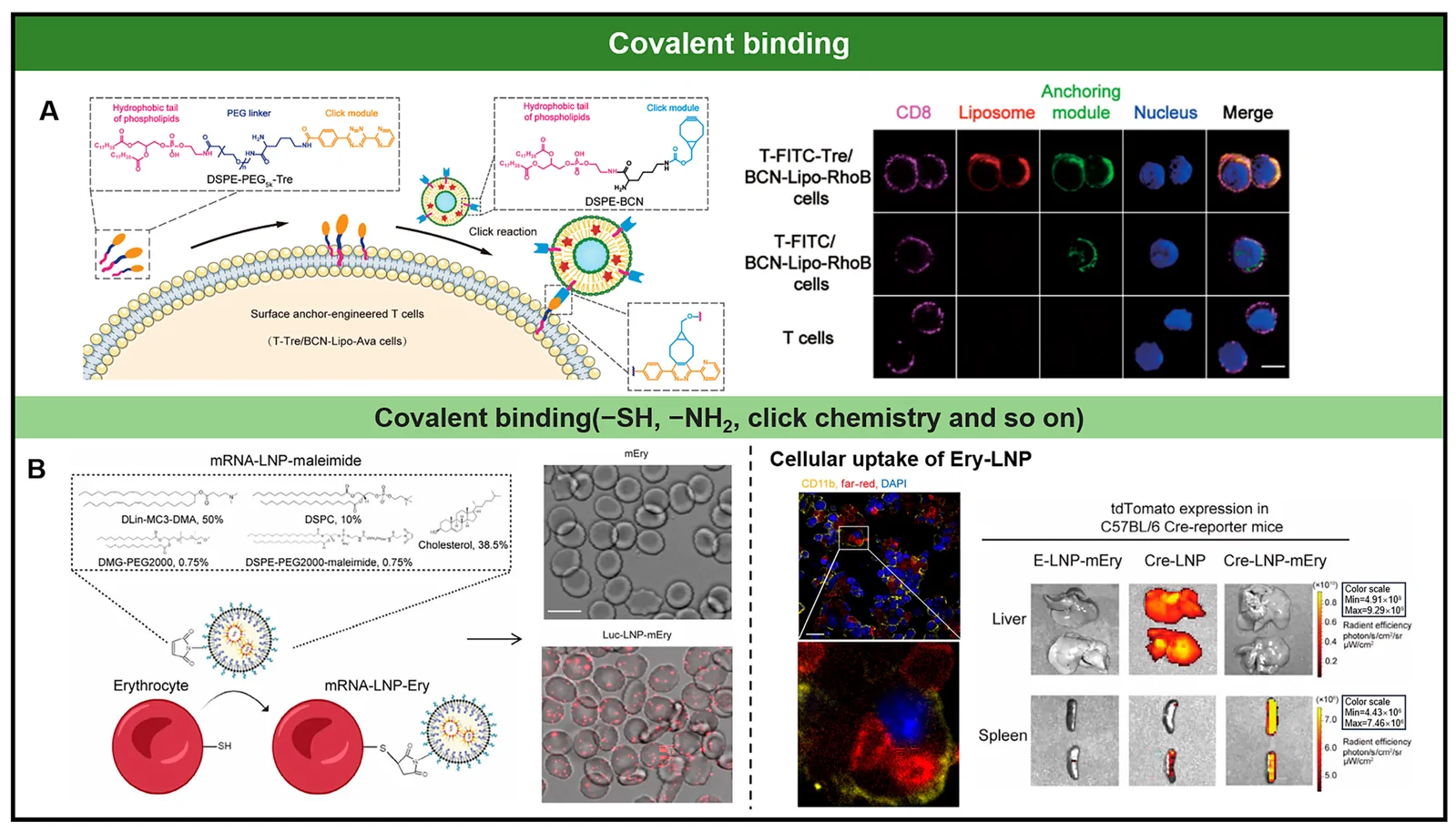
Common strategies for covalent binding of NCs. (**A**) Lipid insertion anchors liposomal avasimibe onto T cells, inducing rapid TCR clustering and sustained T-cell activation [140]. Reprinted with permission from {Hao, M.; Hou, S.; Li, W.; Li, K.; Xue, L.; Hu, Q.; Zhu, L.; Chen, Y.; Sun, H.; Ju, C.; et al. Combination of Metabolic Intervention and T Cell Therapy Enhances Solid Tumor Immunotherapy. *Sci. Transl. Med.* **2020**, *12*, eaaz6667.} Copyright {2020} American Association for the Advancement of Science (AAAS). (**B**) A spleen-targeting system based on red blood cells loaded with mRNA-LNPs, enabling in situ generation of CAR-engineered myeloid cells for antitumor therapy [141]. Reprinted with permission from {Nie, X.; Liu, Y.; Song, Y.; Yao, X.; Chen, Y.; Lee, H.-Y.; Gao, X. In vivo Generation of CAR Myeloid Cells through Erythrocyte-Mediated mRNA Delivery for Cancer Immunotherapy. *Sci. Transl. Med.* **2026**, *18*, eady6730.} Copyright {2026} American Association for the Advancement of Science (AAAS).

As shown in Figure 6, covalent conjugation of cell–nanocarrier systems involves chemical reactions between functional groups on nanocarriers and cell surfaces, such as thiol groups (–SH), primary amines (–NH_2_), carboxyl groups (–COOH) and azide groups (–N_3_), enabling stronger and more stable anchoring [137]. Compared with non-covalent interactions, covalent conjugation provides higher binding strength and better resistance to blood shear forces, allowing nanocarriers to remain attached to cells for a longer period during circulation [138]. However, covalent conjugation also has several limitations. The efficiency of metabolic glycoengineering-based azide labeling largely depends on cellular metabolic activity and incubation time. Different cell types show substantial variations in labeling efficiency and several hours to days of incubation are usually required to achieve sufficient azide density. In addition, chemical modification may alter the native conformation of membrane proteins or block functional domains, thereby affecting cellular functions, such as directional migration and receptor recognition. Some reactive groups may induce immunogenicity and most covalent bonds are irreversible, limiting controlled drug release [139]. In contrast, non-covalent conjugation is simple to perform and causes less damage to cellular functions. However, its relatively weak stability may result in ligand dissociation during circulation. Therefore, the selection of conjugation strategies requires a balance between attachment stability and preservation of cellular activity.

#### 3.3.1. Thiol (–SH)-Mediated Conjugation

Cell membranes are rich in thiol groups and free thiols (–SH) can act as nucleophiles under physiological conditions to participate in various chemical reactions. These thiol groups provide reactive sites for covalent conjugation of nanocarriers [142]. Maleimide groups rapidly react with free thiols under mildly acidic to neutral conditions (pH 6.5–7.5), forming stable thioether bonds. Gao et al. reduced disulfide bonds on erythrocyte surfaces using the reducing agent tris(2-carboxyethyl)phosphine (TCEP). Maleimide-functionalized LNPs were then covalently anchored onto erythrocytes, enabling in situ macrophage reprogramming [141]. DSPE-PEG-maleimide (DSPE-PEG-Mal) is one of the most commonly used functional lipids. By replacing a fraction of DSPE-PEG in LNPs or liposomes with DSPE-PEG-Mal, maleimide-functionalized nanocarriers can be generated. Wayteck et al. developed a T cell-based liposome delivery system. In this system, liposomes were linked to T cells through disulfide bonds formed between thiol groups on T cell membranes and thiol-reactive lipids on liposomes. Approximately 50–60% of liposomes were responsively released from T cells under high glutathione concentrations through thiol–disulfide exchange reactions [143].

#### 3.3.2. Primary Amine (–NH_2_)-Mediated Conjugation

Compared with other reactive groups, amine groups (–NH_2_) are highly abundant on cell surfaces and provide numerous reaction sites for covalent attachment of nanocarriers. The reaction between N-hydroxysuccinimide (NHS), 1-ethyl-3-(3-dimethylaminopropyl)carbodiimide (EDC) and primary amines is one of the most widely used strategies for cell–nanocarrier conjugation [144]. DSPE-PEG-NHS is a commonly used functional lipid. It can insert into the lipid bilayer of cell membranes and expose the NHS ester group on the cell surface. The NHS ester then reacts with primary amines on membrane proteins, enabling covalent attachment of nanocarriers. Alan J. Russell et al. developed an NHS ester-based strategy for erythrocyte surface engineering. They used the homobifunctional crosslinker NHS-PEG-NHS, in which one NHS ester group reacted with primary amines on erythrocyte surface proteins, while the other reacted with lysine residues of protein A (SpA). This approach enabled SpA display on erythrocyte surfaces and facilitated efficient binding of immunoglobulins [145].

#### 3.3.3. Bioorthogonal Click Chemistry

Bioorthogonal conjugation relies on non-native reactive groups introduced onto the surfaces of both cells and nanocarriers. These groups undergo highly selective reactions in complex biological environments, enabling covalent coupling while minimizing nonspecific modification of membrane proteins. One of the most widely used approaches combines metabolic glycoengineering with copper-free click chemistry. The first step involves incubating cells with azide-bearing monosaccharide derivatives, such as tetraacetylated N-azidoacetylmannosamine (Ac_4_ManNAz). Through the endogenous sialic acid biosynthetic pathway, the resulting azide-containing sialic acids are metabolically incorporated into cell-surface glycans, thereby introducing azide groups onto the cell membrane. In the second step, azide-labeled cells are reacted with nanocarriers functionalized with dibenzocyclooctyne (DBCO). Under physiological conditions, the two components undergo strain-promoted azide–alkyne cycloaddition (SPAAC) without the need for a catalyst, forming stable triazole linkages and achieving covalent attachment of nanocarriers to the cell surface [146]. The core advantage of bioorthogonal click chemistry lies in its high targeting specificity and preservation of cell viability. Since non-natural azido sugars are only metabolically incorporated into newly synthesized surface glycans without directly chemically modifying native membrane proteins, cellular biological functions remain well preserved [147]. Li et al. co-incubated Ac_4_ManNAz with T cells to achieve surface azidation, followed by a reaction with DBCO-modified lipid nanoparticles (LNPs), achieving controlled conjugation of LNPs onto T cell surfaces. This system did not affect T cell viability or function and allowed flexible loading of different drug molecules for cell-mediated drug delivery [148].

#### 3.3.4. PEGylation and Linker Modification

PEGylation and linker modification represent a modular strategy for cell–nanodrug conjugation. PEG derivatives containing functional groups are first introduced onto the NCs surface through lipid insertion or polymer modification. The terminal functional groups of PEG, such as NHS ester, maleimide, biotin, or azide groups, can further react with specific groups on the cell membrane through covalent coupling or specific recognition. The PEG linker provides sufficient spatial extension, reducing steric hindrance between NCs and cell membranes. In addition, PEG modification forms a hydrophilic bridge between the NCs surface and affinity ligands, which can reduce recognition and clearance by RES. Resnier et al.engineered lipid nanocapsules using mal-DSPE-PEG. The DSPE moiety facilitated the insertion of the PEG linker into the nanoparticle membrane, while the terminal maleimide group enabled further conjugation with thiolated ligands, allowing stable presentation of functional molecules on the NCs surface [149].

### 3.4. Responsive Modification Strategies

Cell–nanocarrier conjugation systems optimize the PK/PD profiles of drugs and promote their accumulation at target sites. However, efficient intracellular delivery remains critical, as nanocarriers must overcome endosomal barriers and release their cargos into the cytosol to exert therapeutic effects. Designing stimuli-responsive materials based on tumor microenvironmental or intracellular cues represents an effective strategy for controlled drug release (Table 2). Feng et al. conjugated DOX with fatty alcohols of different chain lengths through redox-sensitive disulfide bonds. Among these prodrugs, the hexadecanol-modified DOX prodrug (DSSC16) showed optimal stability and rapid drug release, significantly improving antitumor efficacy in a 4T1 tumor-bearing mouse model [150]. Similarly, Dong et al. developed copper carbonate nanocarriers via a Cu^2+^-mediated biomineralization strategy for co-delivery of glucose oxidase and HIF-α DNAzyme. The pH-responsive dissolution of the mineral framework promoted intracellular cargo release and enhanced cytosolic delivery of macromolecular therapeutics, achieving synergistic antitumor effects through starvation therapy, chemodynamic therapy and gene regulation [151]. Biomacromolecular therapeutics, including proteins, nucleic acids and peptides, often exhibit poor cytosolic delivery efficiency due to structural instability and limited permeability across biological barriers. Various strategies, including cell-penetrating peptides (CPPs), metal ion-mediated systems [152], self-assembled protein nanoparticles [153] and phase separation-based droplets [154], have been developed to improve cytosolic delivery. Wu et al. constructed nanostructures (NAs) by conjugating artesunate (ART) with unsaturated fatty alcohols and modifying their surface with DSPE-SS-PEG2000. Following cellular uptake, NAs achieved triggered cargo release through GSH-mediated disulfide bond cleavage and ROS-triggered oxidation of unsaturated bonds [155]. Wang et al. developed a tea polyphenol-based nanoplatform through oxidative self-polymerization of epigallocatechin gallate (EGCG), enabling efficient protein encapsulation through multiple non-covalent interactions. These NPs facilitated cytosolic protein release and showed significant therapeutic effects in models of hyperuricemia and gouty arthritis [156]. Efficient cytosolic delivery is essential for improving the therapeutic efficacy of anticancer agents [157]. Various nanodelivery strategies, particularly stimuli-responsive nanoplatforms and biomimetic delivery systems, have been developed to enhance cytosolic delivery. However, achieving efficient and precise cytosolic delivery while maintaining biosafety remains a major challenge in cancer nanotherapy.

## 4. Applications and Challenges

### 4.1. Applications in Cancer Therapy

Cancer remains one of the leading causes of death worldwide, posing a major challenge to public health systems due to its high incidence and mortality rates [164,165,166]. Although significant advances have been achieved in cancer treatment, including chemotherapy, radiotherapy, PD-1/PD-L1-based immunotherapy, antibody–drug conjugates (ADCs) and CRISPR/Cas9-mediated gene editing, clinical outcomes are still limited by tumor heterogeneity, complex tumor microenvironments, immune-related adverse effects and the instability of genetic therapeutics [167,168,169]. Immune cells, such as T cells and NK cells, can specifically recognize and eliminate tumor cells. They also possess the ability to actively migrate and infiltrate tumor or inflammatory sites, enabling synergistic effects between drug delivery and immunotherapy. Kim et al. used DSPE-PEG-derived lipids to insert drug-loaded carbon nanotube (CNT–DOX) nanoparticles into the membrane of MSCs [170]. This strategy preserved the migration ability of MSCs, improved drug targeting and enhanced antitumor efficacy. Ning et al. developed engineered macrophages expressing HER2-targeting affibody and carrying DOX-loaded nanoparticles. This system enabled precise drug delivery and effective treatment of HER2-positive breast cancer [171]. Although most cell–nanomedicine conjugation systems remain at the preclinical stage, they show great potential for targeted and precision cancer therapy.

### 4.2. Applications in Immunomodulation

Immune dysregulation is closely associated with various diseases, including cancer, infections, autoimmune disorders and tissue injury. Therefore, immune modulation has become an important therapeutic strategy for these diseases [172,173]. Current immunomodulatory agents, including small-molecule drugs, cytokines and monoclonal antibodies, still face several limitations, such as insufficient targeting, short in vivo retention and off-target toxicity [174,175]. Recently, immune cells have attracted increasing attention as delivery carriers due to their natural homing ability and immunoregulatory functions. Macrophages and neutrophils are among the most widely studied immune cell carriers. Gao et al. anchored drug-loaded NPs onto neutrophil surfaces. By utilizing the natural recruitment of neutrophils to inflammatory and tumor sites, this system achieved targeted treatment of postoperative tumors and promoted immune microenvironment remodeling [176]. Zhou et al. conjugated Poly I-loaded PLGA NPs onto macrophage surfaces. This strategy induced macrophage polarization toward the M1 phenotype and reprogrammed tumor-associated macrophages, thereby enhancing antitumor immune responses [177]. Pu et al. developed a Ly6G antibody-functionalized cerium single-atom catalyst that selectively targeted neutrophils. By exploiting neutrophil-mediated inflammatory homing, this system achieved targeted delivery to acute kidney injury (AKI) sites and effectively alleviated oxidative stress and inflammatory damage. These findings highlight the potential of neutrophil-mediated nanomedicine delivery for inflammatory disease treatment [178]. In the future, immune cell–nanocarrier delivery systems may provide new strategies for remodeling immune microenvironments and enable personalized immune regulation for complex diseases.

### 4.3. Applications in (CNS) Diseases

Central nervous system (CNS) therapy remains challenging due to the restrictive nature of the blood–brain barrier (BBB), which limits drug accumulation and BBB penetration [179]. Mesenchymal stem cells (MSCs) and macrophages possess natural homing abilities and BBB-crossing capacity. They can actively transport nanomedicines to brain lesions while providing immunomodulatory and tissue-repair functions [180]. Monocyte-based NC delivery systems can cross the BBB under neuroinflammatory conditions through CD47–SIRPα-associated cellular interactions and transendothelial migration. These systems enable the delivery of anti-inflammatory agents or neurotrophic factors to pathological regions in the brain [181]. Xue et al. developed a neutrophil-mediated nanomedicine delivery system. By exploiting the rapid recruitment of neutrophils in response to inflammatory signals, this system achieved BBB penetration and significantly inhibited tumor growth in a glioblastoma model, resulting in prolonged survival [105]. Furthermore, Mac et al. developed a chemically programmed prodrug nanoparticle platform based on β-glucan (βGlus–DA(OAc)_2_ NPs). After oral administration, these NPs were taken up by intestinal macrophages and transported through lymphatic and circulatory systems. Guided by inflammatory chemokine gradients, the system crossed the BBB and accumulated in the deep brain substantia nigra region [182]. Therefore, cell-mediated NC delivery strategies, which utilize the intrinsic BBB-crossing ability of living cells, provide a promising approach for CNS disease treatment.

### 4.4. Challenges

#### 4.4.1. Cell Sources and Safety Evaluation

Living cells represent a promising class of drug delivery carriers with unique biological functions beyond conventional nanocarriers. However, the safety and controllability of cell manufacturing remain major challenges (Table 3). Cell sources include autologous, allogeneic and xenogeneic cells [183]. Although autologous cells have low immunogenicity, their clinical application is limited by insufficient cell availability and repeated collection procedures [184]. Allogeneic cells provide a more scalable source but face the challenge of immune rejection. In addition, quality control (QC) and good manufacturing practice (GMP) compliance are critical for cell-based products. Unlike conventional drugs, cellular CQAs are defined not only by purity and quantity but also by potency, phenotype and metabolic state [185]. For example, macrophages exhibit dynamic M0/M1/M2 polarization, while MSCs may develop genomic instability during extensive expansion [186,187]. Moreover, the lack of terminal sterilization requires strict aseptic manufacturing conditions. Future advances in automated manufacturing and intelligent quality control platforms may facilitate the standardization and clinical translation of cell-based therapies.

#### 4.4.2. Storage Stability

Storage stability represents a greater challenge for cell–nanocarrier conjugate delivery systems, as the stability of both living cells and nanocarriers must be simultaneously maintained. Living cells are highly sensitive to temperature changes, osmotic stress and mechanical stimuli. During cryopreservation and thawing, ice crystal formation, membrane damage and metabolic disturbances may occur, leading to reduced cell viability, proliferation and chemotactic ability [188]. Cells with short circulation half-lives, such as neutrophils (~7 h), pose additional challenges for storage and nanocarrier modification. Moreover, freeze–thaw processes may cause nanoparticle detachment, drug leakage and reduced conjugation stability [189]. Therefore, the development of cryoprotective strategies that preserve both cellular function and conjugate stability, together with comprehensive quality monitoring throughout the manufacturing process, is essential for the scalable production and clinical translation of cell-based therapeutics.

#### 4.4.3. Cell–Nanocarrier Compatibility

The compatibility between cells and nanocarriers requires a balance between drug loading capacity and preservation of cellular functions. Increasing the surface modification density of nanocarriers can theoretically enhance drug loading. However, excessive modification may mask functional receptors on the cell surface, alter membrane fluidity and mechanical properties and consequently impair cell viability and homing ability [190]. For example, increasing nanoparticle concentrations can induce phosphatidylserine (PS) exposure on erythrocyte membranes, leading to hemolysis [191]. In addition, different conjugation strategies may have distinct effects on cellular functions. Covalent attachment of nanocarriers to cell surfaces through maleimide (Mal)–thiol (-SH) chemistry consumes membrane thiol groups and the reduction in available -SH groups may trigger cellular stress responses [192]. Moreover, insertion of DSPE-PEG2000 lipids into erythrocyte membranes can induce echinocyte formation within 24h. Furthermore, the material composition, surface charge and chemical properties of nanocarriers may activate the complement system or innate immune pathways, resulting in inflammatory cytokine release, accelerated immune clearance of cell carriers, shortened circulation time and increased risk of immunotoxicity [193].

#### 4.4.4. Drug Release

Delivering drugs to target tissues is only the first step. Controlled drug release at the disease site is critical for therapeutic efficacy. A major challenge is balancing the stability of cell–nanocarrier conjugates with efficient drug release. Covalent conjugation provides strong attachment but may limit drug release when nanocarriers are tightly anchored to the cell surface. In contrast, non-covalent adsorption is vulnerable to blood shear stress and protein corona interference, leading to premature nanocarrier detachment. Moreover, surface-loaded nanocarriers may not remain associated with cells throughout circulation. For example, macrophages may internalize and degrade nanomedicines, while extracellular vesicle release during MSC migration may contribute to nanoparticle loss [194]. Stimuli-responsive release strategies may provide an effective solution by incorporating linkers that are responsive to tumor- or inflammation-associated signals, such as low pH, reactive oxygen species (ROS) and enzymes, enabling controlled nanocarrier release within the target microenvironment.

### 4.5. Future Directions

Although cell–nanocarrier delivery systems are still in the early research stage, they represent a promising therapeutic strategy for challenging diseases, including cancer. To promote the clinical translation of cell-mediated therapies, several key research directions need to be addressed. First, the stability of cell–nanocarrier interactions is closely related to the in vivo fate of NCs. To achieve precise control over nanomedicine behavior, future studies should investigate the adsorption/desorption kinetics between cells and NCs, NPs attachment efficiency and ex vivo organ-based models for further evaluation. In addition, cell-based products are mainly manufactured ex vivo. Conventional cell–nanocarrier conjugation strategies usually involve cell isolation, in vitro expansion, surface modification and quality control. These procedures increase production costs and may alter cellular states. Therefore, in situ hitchhiking strategies have attracted increasing attention. This approach utilizes endogenous cells as nanomedicine carriers, allowing nanocarriers to directly bind circulating or disease-associated cells within the body. It avoids potential issues caused by ex vivo cell manipulation. However, improving in vivo binding specificity and preventing modification of non-target cells remain major challenges. Second, the development of stimuli-responsive cell–nanocarrier conjugation systems represents another important research direction. Cleavable linkers can be designed based on the unique features of tumor microenvironments, including low pH, elevated reactive oxygen species (ROS) and disease-associated enzyme activity. Examples include ROS-sensitive thioether bonds, MMP-2/MMP-9-responsive peptide linkers and acid-sensitive hydrazone bonds. These strategies enable selective release of nanomedicines at disease sites [195,196,197]. Finally, biomaterial-based cell encapsulation strategies may improve the immunocompatibility and survival of transplanted cells. For example, encapsulation of individual mesenchymal stem cells (MSCs) within ultrathin alginate microgels can enhance cell viability and preserve cellular functions [198]. Moreover, artificial intelligence (AI)-based models may predict optimal cell–nanocarrier ratios, drug loading density, release profiles and biocompatibility, thereby improving the efficiency of cell–nanocarrier system design.

## 5. Conclusions

Cell–nanomedicine conjugation technology combines the unique biological functions of living cells with the drug delivery capacity of nanocarriers, creating a new class of therapeutic platforms with “living delivery” characteristics. Unlike conventional nanocarriers that mainly rely on material design for passive targeting or prolonged circulation, this strategy fully exploits the intrinsic properties of cells, including immune evasion, tissue homing, barrier-crossing ability and microenvironment responsiveness. These features provide nanomedicines with enhanced in vivo transport and disease-targeting capabilities. In recent years, various cell types, including erythrocytes, macrophages, T/NK cells, mesenchymal stem cells (MSCs) and engineered microorganisms, have been explored as nanomedicine carriers. These systems have shown great potential in cancer therapy, inflammatory diseases, neurological disorders and gene therapy.

In terms of construction strategies, the interaction mode between cells and nanocarriers determines the stability, biocompatibility and therapeutic performance of the designed system. Covalent conjugation strategies including maleimide–thiol coupling, NHS–amine coupling and copper-free bioorthogonal click chemistry enable stable and controllable anchoring of nanocarriers onto cell surfaces. These strategies are suitable for delivery systems requiring prolonged circulation or precise drug release. However, excessive chemical modification might interfere with membrane protein functions and cellular physiological behaviors. In contrast, non-covalent strategies are mild and cause limited cellular damage, allowing better preservation of natural cell functions. Nevertheless, their binding stability and resistance to shear forces in vivo require further improvement. Therefore, future conjugation strategies should achieve a dynamic balance between binding strength, drug accessibility and preservation of cellular functions.

Although cell–nanocarrier conjugation systems show significant advantages in improving drug delivery efficiency, overcoming biological barriers and enhancing disease targeting, their clinical translation still faces several challenges. The formation of protein coronas, nanocarrier detachment caused by blood shear forces, cellular metabolism and immune clearance might compromise the integrity and delivery efficiency of cell–nanocarrier complexes. In addition, the biological characteristics of cell carriers themselves may limit their applications. For example, macrophages might undergo M1/M2 phenotype switching in the tumor microenvironment. MSCs might have potential tumor-promoting effects and immune cells could be affected by functional exhaustion, excessive activation or limited in vivo persistence. Therefore, systematic evaluation of cell status, immune safety and long-term biological effects are required while improving delivery efficiency.

Future studies should further enhance the controllability and clinical applicability of cell–nanocarrier conjugation systems. On one hand, the development of cleavable linkers and responsive nanomaterials could enable controlled drug release at target tissues while reducing premature detachment and nonspecific exposure during circulation. On the other hand, genetic engineering and synthetic biology approaches might provide new opportunities to regulate cellular functions and enhance disease recognition and therapeutic responses. Overall, cell–nanocarrier conjugation technology represents an important transition in nanomedicine from material-driven design toward biologically functional delivery. By integrating the natural transport capabilities of cells with the tunability of nanotechnology, this strategy provides a promising pathway for next-generation precision drug delivery and personalized therapy.

## Figures and Tables

**Table 1:** Comparative summary of cellular drug carriers.

Carriers	Drug-Loading Capacity	Circulation Half-Life In Vivo	Primary Targeting Mechanism	Major Clinical/ Immunological Risks
Erythrocytes	High	120 days	CD47 downregulation and Ps exposure; MPS-mediated splenic clearance	Blood incompatibility; Low immunogenicity
Platelets	High loading capacity on the cell surface	7–10 days	P-selection binds to ligands PSGL-1 and CD44	The high risk of premature activation/thrombosis; Adheres to circulating tumor cells and promotes tumor metastasis by secretion of TGF-β
Macrophages	High	Several months	Chemokine–chemokine receptor axis	Possibility of Intracellular drug degradation; Macrophage phenotypic plasticity
T cells	Relatively low	4–35 days	Chemokine–chemokine receptor axis	CRS; ICANS; GVHD
NK cells	Relatively low	~14 days	Chemokine–chemokine receptor axis	Immune activation
MSCs	High	~24 h	CXCR4–SDF-1 (CXCL12) chemokine axis	May promote tumor progression; Unpredictable differentiation
Bacteria	Relatively high	No fixed time	Chemotaxis; Tumor microenvironment tropism	Infection; Endotoxin toxicity

**Table 2:** Summary of robust and responsive conjugation methods for cell-based nanocarrier construction.

Loading Strategy	Interaction Pathways	Cell Carrier	Applications	Comment	Ref
Encapsulation	Hypotonic dialysis method	Erythrocytes	Epilepsy	High drug-loading capacity; Potential degradation of NCs after macrophage uptake	[115]
Non-Covalent Conjugation	Ligand–receptor-mediated	Macrophages	Cancer; Inflammatory diseases	Mild and easy to operate; Prone to detachment during blood circulation	[158]
	Nonspecific Adsorption	Macrophages	Cancer		[159,160]
	Host–guest molecular interaction	Erythrocytes	Specific therapy of acute pneumonia		[136]
Covalent Conjugation	Thiol–maleimide conjugation	Macrophages	Cancer	Strong binding; Complex preparation; Difficult to achieve controlled release	[161]
	Bioorthogonal Click Chemistry	T cells	-		[162]
Responsive Modification Strategies	ROS-responsive thioketal (TK) bond	Neutrophils	Cerebral ischemia–reperfusion injury.	Controlled release; Enhanced cytosolic delivery	[163]
	GSH-responsive -S-S- bond	T cells	Cancer		[143]

**Table 3:** Critical challenges for clinical translation.

Challenge	Key Issues	Consequences
Cell source and manufacturing	Autologous call scarcity; Allogeneic immune rejection; Stringent GMP/QC requirements.	High manufacturing cost; Safety and consistency hard to ensure
Storage stability	Cell viability hard to maintain; Nanocarriers detachment.	Decreased cell viability; Reduced homing ability; Lowered conjugation stability
Cell–nanocarrier compatibility	High loading effects on receptors and membrane; Nanomaterials trigger complement immunity.	Decreased cell migration/homing; Increased immunotoxicity
Controlled drug release	Stability vs. efficiency trade-off; Release precision limitation.	Premature drug leakage or insufficient drug release

## Data Availability

No new data were created or analyzed in this study. Data sharing is not applicable to this article.

## References

[1] Fan D., Cao Y., Cao M., Wang Y., Cao Y., Gong T. (2023). Nanomedicine in Cancer Therapy. Signal Transduct. Target. Ther..

[2] Shi R., Fu J., Li M., Zhang A., Huang S., Yang X., Liang Y., Quan Y., He S., Huang S. (2025). Monocyte Membrane-Coated Drug Nanocrystals for Enhanced Targeted Therapy of Rheumatoid Arthritis. J. Control. Release.

[3] He S., Wang L., Wu D., Tong F., Zhao H., Li H., Gong T., Gao H., Zhou Y. (2024). Dual-Responsive Supramolecular Photodynamic Nanomedicine with Activatable Immunomodulation for Enhanced Antitumor Therapy. Acta Pharm. Sin. B.

[4] Zuo Q., Lyu J., Shen X., Wang F., Xing L., Zhou M., Zhou Z., Li L., Huang Y. (2024). A Less-Is-More Strategy for Mitochondria-Targeted Photodynamic Therapy of Rheumatoid Arthritis. Small.

[5] Sung H., Ferlay J., Siegel R.L., Laversanne M., Soerjomataram I., Jemal A., Bray F. (2021). Global Cancer Statistics 2020: GLOBOCAN Estimates of Incidence and Mortality Worldwide for 36 Cancers in 185 Countries. CA Cancer J. Clin..

[6] Pardridge W.M. (2019). Blood-Brain Barrier and Delivery of Protein and Gene Therapeutics to Brain. Front. Aging Neurosci..

[7] Jiang C., Yang X., Huang Q., Lei T., Luo H., Wu D., Yang Z., Xu Y., Dou Y., Ma X. (2025). Microglial-Biomimetic Memantine-Loaded Polydopamine Nanomedicines for Alleviating Depression. Adv. Mater..

[8] Zhou M., Luo C., Zhou Z., Li L., Huang Y. (2021). Improving Anti-PD-L1 Therapy in Triple Negative Breast Cancer by Polymer-Enhanced Immunogenic Cell Death and CXCR4 Blockade. J. Control. Release.

[9] Jia N., Gao Y., Li M., Liang Y., Li Y., Lin Y., Huang S., Lin Q., Sun X., He Q. (2023). Metabolic Reprogramming of Proinflammatory Macrophages by Target Delivered Roburic Acid Effectively Ameliorates Rheumatoid Arthritis Symptoms. Signal Transduct. Target. Ther..

[10] Liu C., Li L., Lyu J., Xiang Y., Chen L., Zhou Z., Huang Y. (2022). Split Bullets Loaded Nanoparticles for Amplified Immunotherapy. J. Control. Release.

[11] Yan Y., Li J., Yi X., Liu C., Zhou Z., Huang Y., Li L. (2022). Peritumoral Scaffold Neutralizes Tumor pH for Chemotherapy Sensitization and Metastasis Inhibition. J. Control. Release.

[12] Tao J., Yao Y., Huang M., Wu J., Lyu J., Li Q., Li L., Huang Y., Zhou Z. (2024). A Nano-Platform Combats the “Attack” and “Defense” of Cytoskeleton to Block Cascading Tumor Metastasis. J. Control. Release.

[13] Zhou M., Liu C., Li B., Li J., Zhang P., Huang Y., Li L. (2024). Cell Surface Patching via CXCR4-Targeted Nanothreads for Cancer Metastasis Inhibition. Nat. Commun..

[14] Gabizon A.A., Gabizon-Peretz S., Modaresahmadi S., La-Beck N.M. (2025). Thirty Years from FDA Approval of Pegylated Liposomal Doxorubicin (Doxil/Caelyx): An Updated Analysis and Future Perspective. BMJ Oncol..

[15] Kadri H., Alshatfa M., Alsalloum F.Z., Elhissi A., Daou A., Khoder M. (2025). Albumin Nanoparticles in Cancer Therapeutics: Clinical Status, Challenges, and Future Directions. Pharmaceutics.

[16] Adams D., Gonzalez-Duarte A., O’Riordan W.D., Yang C.-C., Ueda M., Kristen A.V., Tournev I., Schmidt H.H., Coelho T., Berk J.L. (2018). Patisiran, an RNAi Therapeutic, for Hereditary Transthyretin Amyloidosis. N. Engl. J. Med..

[17] Wang Q., Xia X., Zhang H., Li Y., Zhu L., Shi Y., Tang Y., Cheng J., Hui X., Gao H. (2025). Targeting Modulation of the Choroid Plexus Blood-CSF Barrier and CSF Hypersecretion via Lipid Nanoparticle-Mediated Co-Delivery of siRNA and Resveratrol. Nat. Commun..

[18] Yang X., Yang W., Xia X., Lei T., Yang Z., Jia W., Zhou Y., Cheng G., Gao H. (2022). Intranasal Delivery of BACE1 siRNA and Rapamycin by Dual Targets Modified Nanoparticles for Alzheimer’s Disease Therapy. Small.

[19] Li S., Li L., Lin X., Chen C., Luo C., Huang Y. (2022). Targeted Inhibition of Tumor Inflammation and Tumor-Platelet Crosstalk by Nanoparticle-Mediated Drug Delivery Mitigates Cancer Metastasis. ACS Nano.

[20] Hu C., Song Y., Zhang Y., He S., Liu X., Yang X., Gong T., Huang Y., Gao H. (2023). Sequential Delivery of PD-1/PD-L1 Blockade Peptide and IDO Inhibitor for Immunosuppressive Microenvironment Remodeling via an MMP-2 Responsive Dual-Targeting Liposome. Acta Pharm. Sin. B.

[21] Li J., Yan Y., Zhang P., Ding J., Huang Y., Jin Y., Li L. (2022). A Cell-Laden Hydrogel as Prophylactic Vaccine and Anti-PD-L1 Amplifier against Autologous Tumors. J. Control. Release.

[22] Li M., Chen S., Guo R., Wang Y., Yang M., Liu Y., Zhang Q., Zhu S., Li J., Chen F. (2025). Microglia-Derived Nanovesicles Synchronize Macroautophagy and Chaperone-Mediated Autophagy for Alzheimer’s Disease Therapy. Signal Transduct. Target. Ther..

[23] Bai W., Chen S., Liu T., Tang X., Xiong L., Li M., Guo R., He Q. (2025). Dual-Action Membrane-Chimeric Liposomes with Self-Reinforcing Targeting for Acute Lung Injury Treatment. J. Control. Release.

[24] Ma C., Zhong X., Liu R., Yang X., Xie Z., Zhang Y., Xu Y., Wang H., He C., Du G. (2024). Co-Delivery of Oxaliplatin Prodrug Liposomes with Bacillus Calmette-Guérin for Chemo-Immunotherapy of Orthotopic Bladder Cancer. J. Control. Release.

[25] Wu L., Wang L., Liu X., Bai Y., Wu R., Li X., Mao Y., Zhang L., Zheng Y., Gong T. (2022). Milk-Derived Exosomes Exhibit Versatile Effects for Improved Oral Drug Delivery. Acta Pharm. Sin. B.

[26] Sun R., Xiang J., Zhou Q., Piao Y., Tang J., Shao S., Zhou Z., Bae Y.H., Shen Y. (2022). The Tumor EPR Effect for Cancer Drug Delivery: Current Status, Limitations, and Alternatives. Adv. Drug Deliv. Rev..

[27] Chen L., Hong W., Ren W., Xu T., Qian Z., He Z. (2021). Recent Progress in Targeted Delivery Vectors Based on Biomimetic Nanoparticles. Signal Transduct. Target. Ther..

[28] Li L., Zhang M., Li J., Liu T., Bao Q., Li X., Long J., Fu L., Zhang Z., Huang S. (2023). Cholesterol Removal Improves Performance of a Model Biomimetic System to Co-Deliver a Photothermal Agent and a STING Agonist for Cancer Immunotherapy. Nat. Commun..

[29] Liu R., Jia W., Wang Y., Hu C., Yu W., Huang Y., Wang L., Gao H. (2022). Glymphatic System and Subsidiary Pathways Drive Nanoparticles Away from the Brain. Research.

[30] Wang D., Dong H., Li M., Cao Y., Yang F., Zhang K., Dai W., Wang C., Zhang X. (2018). Erythrocyte-Cancer Hybrid Membrane Camouflaged Hollow Copper Sulfide Nanoparticles for Prolonged Circulation Life and Homotypic-Targeting Photothermal/Chemotherapy of Melanoma. ACS Nano.

[31] Hu C., Lei T., Wang Y., Cao J., Yang X., Qin L., Liu R., Zhou Y., Tong F., Umeshappa C.S. (2020). Phagocyte-Membrane-Coated and Laser-Responsive Nanoparticles Control Primary and Metastatic Cancer by Inducing Anti-Tumor Immunity. Biomaterials.

[32] Zang S., Huang K., Li J., Ren K., Li T., He X., Tao Y., He J., Dong Z., Li M. (2022). Metabolic Reprogramming by Dual-Targeting Biomimetic Nanoparticles for Enhanced Tumor Chemo-Immunotherapy. Acta Biomater..

[33] Qin M., Du G., Sun X. (2020). Biomimetic Cell-Derived Nanocarriers for Modulating Immune Responses. Biomater. Sci..

[34] Wang S., Ma S., Li R., Qi X., Han K., Guo L., Li X. (2023). Probing the Interaction Between Supercarrier RBC Membrane and Nanoparticles for Optimal Drug Delivery. J. Mol. Biol..

[35] Bashiri G., Padilla M.S., Swingle K.L., Shepherd S.J., Mitchell M.J., Wang K. (2023). Nanoparticle Protein Corona: From Structure and Function to Therapeutic Targeting. Lab Chip.

[36] Soprano E., Migliavacca M., López-Ferreiro M., Pelaz B., Polo E., Del Pino P. (2023). Fusogenic Cell-Derived Nanocarriers for Cytosolic Delivery of Cargo inside Living Cells. J. Colloid Interface Sci..

[37] Chen Y., Qin D., Zou J., Li X., Guo X.D., Tang Y., Liu C., Chen W., Kong N., Zhang C.Y. (2023). Living Leukocyte-Based Drug Delivery Systems. Adv. Mater..

[38] Zhang F., Xu Z., Jolly K.J. (2023). Myeloid Cell-Mediated Drug Delivery: From Nanomedicine to Cell Therapy. Adv. Drug Deliv. Rev..

[39] Gao C., Wang Q., Li J., Kwong C.H.T., Wei J., Xie B., Lu S., Lee S.M.Y., Wang R. (2022). In Vivo Hitchhiking of Immune Cells by Intracellular Self-Assembly of Bacteria-Mimetic Nanomedicine for Targeted Therapy of Melanoma. Sci. Adv..

[40] Christensen J.R., Reck-Peterson S.L. (2022). Hitchhiking Across Kingdoms: Cotransport of Cargos in Fungal, Animal, and Plant Cells. Annu. Rev. Cell Dev. Biol..

[41] Zhang S., Fu Q., Zhang Y., Pan J., Zhang L., Zhang Z., Liu Z. (2021). Surface Loading of Nanoparticles on Engineered or Natural Erythrocytes for Prolonged Circulation Time: Strategies and Applications. Acta Pharmacol. Sin..

[42] Anselmo A.C., Gupta V., Zern B.J., Pan D., Zakrewsky M., Muzykantov V., Mitragotri S. (2013). Delivering Nanoparticles to Lungs While Avoiding Liver and Spleen through Adsorption on Red Blood Cells. ACS Nano.

[43] Brenner J.S., Pan D.C., Myerson J.W., Marcos-Contreras O.A., Villa C.H., Patel P., Hekierski H., Chatterjee S., Tao J.-Q., Parhiz H. (2018). Red Blood Cell-Hitchhiking Boosts Delivery of Nanocarriers to Chosen Organs by Orders of Magnitude. Nat. Commun..

[44] Xu X., Xu Y., Li Y., Li M., Wang L., Zhang Q., Zhou B., Lin Q., Gong T., Sun X. (2022). Glucose-Responsive Erythrocyte-Bound Nanoparticles for Continuously Modulated Insulin Release. Nano Res..

[45] Shan L., Wang J., Tu H., Zhang W., Li H., Slezak P., Lu F., Lee D., Hu E., Geng Z. (2024). Drug Delivery under Cover of Erythrocytes Extends Drug Half-Life: A Thrombolytic Targeting Therapy Utilizing Microenvironment-Responsive Artificial Polysaccharide Microvesicles. Carbohydr. Polym..

[46] Garcia-Herreros A., Yeh Y.-T., Peng Z., del Álamo J.C. (2022). Cyclic Mechanical Stresses Alter Erythrocyte Membrane Composition and Microstructure and Trigger Macrophage Phagocytosis. Adv. Sci..

[47] Liu J., Wang F., Li L. (2026). Erythrocyte Patch for Enhanced B Cell Depletion Therapy. Sci. Adv..

[48] Ullah I., Ullah I., Bedros A.W.S., Kim D.H., Kim J.W., Shah S.D., Singh T., Aminy H., Olatunji A.O., Alam N. (2024). Revolutionary Advances in Red Blood Cell-Based Nano carrier Drug Delivery Systems for Specific Tumor Therapy: Progresses, Challenges, and Research Directions. J. Biotechnol. Biomed..

[49] Jubeli E., Moine L., Vergnaud-Gauduchon J., Barratt G. (2012). E-Selectin as a Target for Drug Delivery and Molecular Imaging. J. Control. Release.

[50] Guo R., Deng M., He X., Li M., Li J., He P., Liu H., Li M., Zhang Z., He Q. (2022). Fucoidan-Functionalized Activated Platelet-Hitchhiking Micelles Simultaneously Track Tumor Cells and Remodel the Immunosuppressive Microenvironment for Efficient Metastatic Cancer Treatment. Acta Pharm. Sin. B.

[51] Koupenova M., Clancy L., Corkrey H.A., Freedman J.E. (2018). Circulating Platelets as Mediators of Immunity, Inflammation, and Thrombosis. Circ. Res..

[52] Wang M., Yu Z., Li X., Li J., Li J., Luo J., Li J., Xiong Y., Yang J. (2025). In Situ Dual-Targeted Drug Delivery System for Alleviating Imaging and Pathological Damage in Septic Arthritis. Acta Biomater..

[53] Zhu Y., Xu L., Kang Y., Cheng Q., He Y., Ji X. (2024). Platelet-Derived Drug Delivery Systems: Pioneering Treatment for Cancer, Cardiovascular Diseases, Infectious Diseases, and Beyond. Biomaterials.

[54] Gan J., Zhang X., Guo J. (2025). The Role of Platelets in Tumor Immune Evasion and Metastasis: Mechanisms and Therapeutic Implications. Cancer Cell Int..

[55] Elaskalani O., Berndt M.C., Falasca M., Metharom P. (2017). Targeting Platelets for the Treatment of Cancer. Cancers.

[56] Zhou L., Zhang Z., Tian Y., Li Z., Liu Z., Zhu S. (2023). The Critical Role of Platelet in Cancer Progression and Metastasis. Eur. J. Med. Res..

[57] Ding S., Dong X., Song X. (2023). Tumor Educated Platelet: The Novel BioSource for Cancer Detection. Cancer Cell Int..

[58] Jansen E.E., Braun A., Jansen P., Hartmann M. (2021). Platelet-Therapeutics to Improve Tissue Regeneration and Wound Healing-Physiological Background and Methods of Preparation. Biomedicines.

[59] Wang H., Hu H., Li Y., Hu L., Gu C., Zhu Y., Huang J., Li N., Jiang S., Zu S. (2025). A Conjugation Delivery System of Macrophages and Platelet Pharmacytes Promotes Regeneration After Spinal Cord Injury. Adv. Sci..

[60] Guan F., Wang R., Yi Z., Luo P., Liu W., Xie Y., Liu Z., Xia Z., Zhang H., Cheng Q. (2025). Tissue Macrophages: Origin, Heterogenity, Biological Functions, Diseases and Therapeutic Targets. Signal Transduct. Target. Ther..

[61] Ghamangiz S., Jafari A., Maleki-Kakelar H., Azimi H., Mazloomi E. (2025). Reprogram to Heal: Macrophage Phenotypes as Living Therapeutics. Life Sci..

[62] Xiao M., Li X. (2025). The Impact of the Tumor Microenvironment on Macrophages. Front. Immunol..

[63] Yang H., Zhang Q., Xu M., Wang L., Chen X., Feng Y., Li Y., Zhang X., Cui W., Jia X. (2020). CCL2-CCR2 Axis Recruits Tumor Associated Macrophages to Induce Immune Evasion through PD-1 Signaling in Esophageal Carcinogenesis. Mol. Cancer.

[64] Beider K., Bitner H., Leiba M., Gutwein O., Koren-Michowitz M., Ostrovsky O., Abraham M., Wald H., Galun E., Peled A. (2014). Multiple Myeloma Cells Recruit Tumor-Supportive Macrophages through the CXCR4/CXCL12 Axis and Promote Their Polarization toward the M2 Phenotype. Oncotarget.

[65] Mempel T.R., Lill J.K., Altenburger L.M. (2024). How Chemokines Organize the Tumour Microenvironment. Nat. Rev. Cancer.

[66] Hu C., Wang J., Gao X., Xia J., Li W., Song P., Zhang W., Ge F., Zhu L. (2024). Pluronic-Based Nanoparticles for Delivery of Doxorubicin to the Tumor Microenvironment by Binding to Macrophages. ACS Nano.

[67] Sun L., Kees T., Almeida A.S., Liu B., He X.-Y., Ng D., Han X., Spector D.L., McNeish I.A., Gimotty P. (2021). Activating a Collaborative Innate-Adaptive Immune Response to Control Metastasis. Cancer Cell.

[68] Qian B.-Z., Pollard J.W. (2010). Macrophage Diversity Enhances Tumor Progression and Metastasis. Cell.

[69] Chen S., Li Y., Zhou Z., Saiding Q., Zhang Y., An S., Khan M.M., Ji X., Qiao R., Tao W. (2025). Macrophage Hitchhiking Nanomedicine for Enhanced β-Elemene Delivery and Tumor Therapy. Sci. Adv..

[70] LiYang L., Zhang Y., Zhang Y., Xu Y., Li Y., Xie Z., Wang H., Lin Y., Lin Q., Gong T. (2022). Live Macrophage-Delivered Doxorubicin-Loaded Liposomes Effectively Treat Triple-Negative Breast Cancer. ACS Nano.

[71] Lin Z., Zou S., Wen K. (2024). The Crosstalk of CD8+ T Cells and Ferroptosis in Cancer. Front. Immunol..

[72] Lee N.K., Kim S.-N., Park C.G. (2021). Immune Cell Targeting Nanoparticles: A Review. Biomater. Res..

[73] Huang B., Abraham W.D., Zheng Y., Bustamante López S.C., Luo S.S., Irvine D.J. (2015). Active Targeting of Chemotherapy to Disseminated Tumors Using Nanoparticle-Carrying T Cells. Sci. Transl. Med..

[74] Nayer B., Tan J.L., Alshoubaki Y.K., Lu Y.-Z., Legrand J.M.D., Lau S., Hu N., Park A.J., Wang X.-N., Amann-Zalcenstein D. (2024). Local Administration of Regulatory T Cells Promotes Tissue Healing. Nat. Commun..

[75] Simic M.S., Watchmaker P.B., Gupta S., Wang Y., Sagan S.A., Duecker J., Shepherd C., Diebold D., Pineo-Cavanaugh P., Haegelin J. (2024). Programming Tissue-Sensing T Cells That Deliver Therapies to the Brain. Science.

[76] Wolf N.K., Kissiov D.U., Raulet D.H. (2023). Roles of Natural Killer Cells in Immunity to Cancer, and Applications to Immunotherapy. Nat. Rev. Immunol..

[77] Vivier E., Raulet D.H., Moretta A., Caligiuri M.A., Zitvogel L., Lanier L.L., Yokoyama W.M., Ugolini S. (2011). Innate or Adaptive Immunity? The Example of Natural Killer Cells. Science.

[78] Gauthier L., Morel A., Anceriz N., Rossi B., Blanchard-Alvarez A., Grondin G., Trichard S., Cesari C., Sapet M., Bosco F. (2019). Multifunctional Natural Killer Cell Engagers Targeting NKp46 Trigger Protective Tumor Immunity. Cell.

[79] Nieto Y., Banerjee P., Kaur I., Basar R., Li Y., Daher M., Rafei H., Kerbauy L.N., Kaplan M., Marin D. (2025). Allogeneic NK Cells with a Bispecific Innate Cell Engager in Refractory Relapsed Lymphoma: A Phase 1 Trial. Nat. Med..

[80] Laskowski T.J., Biederstädt A., Rezvani K. (2022). Natural Killer Cells in Antitumour Adoptive Cell Immunotherapy. Nat. Rev. Cancer.

[81] Dai Y., Ding Q., Chen Z., Jiao G., Yang Y., Fu S., Yang Z., Liu X., Qian K., Cheng Z. (2026). NIR-II Nanomedicine Engineered CAR-NK Cells for Precision Navigation and Potentiating Lung Cancer Immunotherapy by Remodeling Tumor Microenvironment. J. Am. Chem. Soc..

[82] Li J., Huang Y., Sun H., Yang L. (2023). Mechanism of Mesenchymal Stem Cells and Exosomes in the Treatment of Age-Related Diseases. Front. Immunol..

[83] Spees J.L., Lee R.H., Gregory C.A. (2016). Mechanisms of Mesenchymal Stem/Stromal Cell Function. Stem Cell Res. Ther..

[84] Zhou L., Song Q., Zhang X., Cao M., Xue D., Sun Y., Mao M., Li X., Zhang Z., Liu J. (2025). In Vivo Generation of CAR Macrophages via the Enucleated Mesenchymal Stem Cell Delivery System for Glioblastoma Therapy. Proc. Natl. Acad. Sci. USA.

[85] Li D., Yang Y., Zheng G., Meng L., Shang L., Ren J., Wang L., Bao Y. (2024). The Potential of Cellular Homing Behavior in Tumor Immunotherapy: From Basic Discoveries to Clinical Applications of Immune, Mesenchymal Stem, and Cancer Cell Homing. Front. Immunol..

[86] Jiang Q., Huang K., Lu F., Deng S., Yang Z., Hu S. (2022). Modifying Strategies for SDF-1/CXCR4 Interaction during Mesenchymal Stem Cell Transplantation. Gen. Thorac. Cardiovasc. Surg..

[87] Zhang T., Lin R., Wu H., Jiang X., Gao J. (2022). Mesenchymal Stem Cells: A Living Carrier for Active Tumor-Targeted Delivery. Adv. Drug Deliv. Rev..

[88] Merino J.J., Cabaña-Muñoz M.E. (2023). Nanoparticles and Mesenchymal Stem Cell (MSC) Therapy for Cancer Treatment: Focus on Nanocarriers and a Si-RNA CXCR4 Chemokine Blocker as Strategies for Tumor Eradication In Vitro and In Vivo. Micromachines.

[89] Minev T., Balbuena S., Gill J.M., Marincola F.M., Kesari S., Lin F. (2024). Mesenchymal Stem Cells—The Secret Agents of Cancer Immunotherapy: Promises, Challenges, and Surprising Twists. Oncotarget.

[90] Wang H., Alarcón C.N., Liu B., Watson F., Searles S., Lee C.K., Keys J., Pi W., Allen D., Lammerding J. (2022). Genetically Engineered and Enucleated Human Mesenchymal Stromal Cells for the Targeted Delivery of Therapeutics to Diseased Tissue. Nat. Biomed. Eng..

[91] Chen W., Zhu Y., Zhang Z., Sun X. (2022). Advances in *Salmonella* Typhimurium-based drug delivery system for cancer therapy. Adv. Drug Deliv. Rev..

[92] Zhang X., Chen G., Zhang H., Shang L., Zhao Y. (2023). Bioinspired Oral Delivery Devices. Nat. Rev. Bioeng..

[93] Li M., Lu M., Lai Y., Zhang X., Li Y., Mao P., Liang Z., Mu Y., Lin Y., Zhao A.Z. (2020). Inhibition of Acute Leukemia with Attenuated *Salmonella typhimurium* Strain VNP20009. Biomed. Pharmacother..

[94] Nanoparticle-Based Targeted Delivery Unleashes the Full Power of Anti-Cancer Drugs. https://phys.org/news/2025-07-nanoparticle-based-delivery-unleashes-full.html.

[95] Liu G., Ma N., Cheng K., Feng Q., Ma X., Yue Y., Li Y., Zhang T., Gao X., Liang J. (2024). Bacteria-Derived Nanovesicles Enhance Tumour Vaccination by Trained Immunity. Nat. Nanotechnol..

[96] Canale F.P., Basso C., Antonini G., Perotti M., Li N., Sokolovska A., Neumann J., James M.J., Geiger S., Jin W. (2021). Metabolic Modulation of Tumours with Engineered Bacteria for Immunotherapy. Nature.

[97] Forbes N.S. (2010). Engineering the Perfect (Bacterial) Cancer Therapy. Nat. Rev. Cancer.

[98] Taniguchi S. (2021). In Situ Delivery and Production System (iDPS) of Anti-Cancer Molecules with Gene-Engineered Bifidobacterium. J. Pers. Med..

[99] Liu W., Cheng G., Cui H., Tian Z., Li B., Han Y., Wu J.-X., Sun J., Zhao Y., Chen T. (2024). Theoretical basis, state and challenges of living cell-based drug delivery systems. Theranostics.

[100] Rennick J.J., Johnston A.P.R., Parton R.G. (2021). Key Principles and Methods for Studying the Endocytosis of Biological and Nanoparticle Therapeutics. Nat. Nanotechnol..

[101] Mondal N., Dalal D.C. (2023). Modelling of Reversible Tissue Electroporation and Its Thermal Effects in Drug Delivery. Eur. J. Pharm. Biopharm..

[102] Wen Z., Liu C., Teng Z., Jin Q., Liao Z., Zhu X., Huo S. (2023). Ultrasound Meets the Cell Membrane: For Enhanced Endocytosis and Drug Delivery. Nanoscale.

[103] Bourgeaux V., Lanao J.M., Bax B.E., Godfrin Y. (2016). Drug-Loaded Erythrocytes: On the Road toward Marketing Approval. Drug Des. Devel. Ther..

[104] Sun P., Deng Q., Kang L., Sun Y., Ren J., Qu X.A. (2020). Smart Nanoparticle-Laden and Remote-Controlled Self-Destructive Macrophage for Enhanced Chemo/Chemodynamic Synergistic Therapy. ACS Nano.

[105] Xue J., Zhao Z., Zhang L., Xue L., Shen S., Wen Y., Wei Z., Wang L., Kong L., Sun H. (2017). Neutrophil-Mediated Anticancer Drug Delivery for Suppression of Postoperative Malignant Glioma Recurrence. Nat. Nanotechnol..

[106] Wu J., Ni M., Xing L., Wang Y., Liu X., Zheng Y., Li L., Huang Y. (2026). Surface Hydrophobicity and Rigidity Determines Protein Corona on Orally Delivered Nanoparticles Treating Colitis. Nat. Commun..

[107] Aderem A., Underhill D.M. (1999). Mechanisms of Phagocytosis in Macrophages. Annu. Rev. Immunol..

[108] Liebl D., Qi X., Zhe Y., Barnett T.C., Teasdale R.D. (2017). SopB-Mediated Recruitment of SNX18 Facilitates *Salmonella* Typhimurium Internalization by the Host Cell. Front. Cell. Infect. Microbiol..

[109] Xie Z., Su Y., Kim G.B., Selvi E., Ma C., Aragon-Sanabria V., Hsieh J.-T., Dong C., Yang J. (2017). Immune Cell-Mediated Biodegradable Theranostic Nanoparticles for Melanoma Targeting and Drug Delivery. Small.

[110] Wang S., Liu J., Cui Y., Sun M., Wang W., Chen J., Gu J., Yang Z. (2025). Macrophage-Centered Therapy Strategies: A Promising Weapon in Cancer Immunotherapy. Asian J. Pharm. Sci..

[111] Ge D., Zou L., Li C., Liu S., Li S., Sun S., Ding W. (2018). Simulation of the Osmosis-Based Drug Encapsulation in Erythrocytes. Eur. Biophys. J..

[112] Bax B.E. (2020). Erythrocytes as Carriers of Therapeutic Enzymes. Pharmaceutics.

[113] Bai S., Hou S., Chen T., Ma X., Lin J., Gao C., Wu A. (2026). Osmolarity-Directed Encapsulation of Size-Tuned Nanoparticles into Red Blood Cells. Small Methods.

[114] Gutierrez-Millan C., Barez Diaz C., Alvarez Vizan L., Colino C.I. (2023). Evaluation of Two Osmosis-Based Methods for the Preparation of Drug Delivery Systems Based on Red Blood Cells. Pharmaceutics.

[115] Hamidi M., Rafiei P., Azadi A., Mohammadi-Samani S. (2011). Encapsulation of Valproate-Loaded Hydrogel Nanoparticles in Intact Human Erythrocytes: A Novel Nano-Cell Composite for Drug Delivery. J. Pharm. Sci..

[116] Ukidve A., Zhao Z., Fehnel A., Krishnan V., Pan D.C., Gao Y., Mandal A., Muzykantov V., Mitragotri S. (2020). Erythrocyte-Driven Immunization via Biomimicry of Their Natural Antigen-Presenting Function. Proc. Natl. Acad. Sci. USA.

[117] Cheng H., Kastrup C.J., Ramanathan R., Siegwart D.J., Ma M., Bogatyrev S.R., Xu Q., Whitehead K.A., Langer R., Anderson D.G. (2010). Nanoparticulate Cellular Patches for Cell-Mediated Tumoritropic Delivery. ACS Nano.

[118] Banerjee T., Biswas D., Pal D.S., Miao Y., Iglesias P.A., Devreotes P.N. (2022). Spatiotemporal Dynamics of Membrane Surface Charge Regulates Cell Polarity and Migration. Nat. Cell Biol..

[119] Issler T., Turner R.J., Prenner E.J. (2024). Membrane-Nanoparticle Interactions: The Impact of Membrane Lipids. Small.

[120] Zhao Z., Ukidve A., Gao Y., Kim J., Mitragotri S. (2019). Erythrocyte Leveraged Chemotherapy (ELeCt): Nanoparticle Assembly on Erythrocyte Surface to Combat Lung Metastasis. Sci. Adv..

[121] Hamadani C.M., Taylor G.R., Cecil A., Chism C.M., Huff E., Dasanayake G., Everett J., Kaur A., Monroe G.W., Patel M. (2025). Insights into the Physicochemical Interactions of Ionic Liquid-Coated Polymeric Nanoparticles with Red Blood Cells. Nanoscale Adv..

[122] Cao M., Fay F., Hotier A., Domenichini S., Alexandre L., Ribes C., Gazeau F., Hillaireau H., Fattal E. (2026). Selective mRNA Delivery to Activated Macrophages via Hyaluronic Acid-Functionalized Lipid Nanoparticles with Optimized PEGylation. Biomacromolecules.

[123] Hlaing S.P., Cao J., Lee J., Kim J., Saparbayeva A., Kwak D., Kim H., Hwang S., Yun H., Moon H.R. (2022). Hyaluronic Acid-Conjugated PLGA Nanoparticles Alleviate Ulcerative Colitis via CD44-Mediated Dual Targeting to Inflamed Colitis Tissue and Macrophages. Pharmaceutics.

[124] Lupoae M., Elisei A.M., Iacob A., Lupoae A., Tatu A.L., Niculeț E., Căuș M.N., Batîr D., Nechita A., Matei M.N. (2026). Cutaneous-Tropism Viruses: Unraveling Pathogenetic Mechanisms and Immunoprophylactic Strategies. Life.

[125] Tang L., Zheng Y., Melo M.B., Mabardi L., Castaño A.P., Xie Y.-Q., Li N., Kudchodkar S.B., Wong H.C., Jeng E.K. (2018). Enhancing T Cell Therapy through TCR-Signaling-Responsive Nanoparticle Drug Delivery. Nat. Biotechnol..

[126] Radford D.C., Yang J., Doan M.C., Li L., Dixon A.S., Owen S.C., Kopeček J. (2020). Multivalent HER2-Binding Polymer Conjugates Facilitate Rapid Endocytosis and Enhance Intracellular Drug Delivery. J. Control. Release.

[127] Jain A., Cheng K. (2017). The Principles and Applications of Avidin-Based Nanoparticles in Drug Delivery and Diagnosis. J. Control. Release.

[128] Ren W.X., Han J., Uhm S., Jang Y.J., Kang C., Kim J.-H., Kim J.S. (2015). Recent Development of Biotin Conjugation in Biological Imaging, Sensing, and Target Delivery. Chem. Commun..

[129] Mooney R., Weng Y., Garcia E., Bhojane S., Smith-Powell L., Kim S.U., Annala A.J., Aboody K.S., Berlin J.M. (2014). Conjugation of pH-responsive nanoparticles to neural stem cells improves intratumoral therapy. J. Control. Release.

[130] Lesch H.P., Kaikkonen M.U., Pikkarainen J.T., Ylä-Herttuala S. (2010). Avidin-Biotin Technology in Targeted Therapy. Expert Opin. Drug Deliv..

[131] Wei Z., Zhou Y., Wang R., Wang J., Chen Z. (2022). Aptamers as Smart Ligands for Targeted Drug Delivery in Cancer Therapy. Pharmaceutics.

[132] Van den Avont A., Sharma-Walia N. (2023). Anti-Nucleolin Aptamer AS1411: An Advancing Therapeutic. Front. Mol. Biosci..

[133] Qi L., Tian Y., Li N., Mao M., Fang X., Han D. (2023). Engineering Circular Aptamer Assemblies with Tunable Selectivity to Cell Membrane Antigens In Vitro and In Vivo. ACS Appl. Mater. Interfaces.

[134] Liu J., Ren Z., Sun Y., Xu L., Wei D., Tan W., Ding D. (2023). Investigation of the Relationship between Aptamers’ Targeting Functions and Human Plasma Proteins. ACS Nano.

[135] Peng L., Liu S., Feng A., Yuan J. (2017). Polymeric Nanocarriers Based on Cyclodextrins for Drug Delivery: Host-Guest Interaction as Stimuli Responsive Linker. Mol. Pharm..

[136] Li J., Ding Y., Cheng Q., Gao C., Wei J., Wang Z., Huang Q., Wang R. (2022). Supramolecular Erythrocytes-Hitchhiking Drug Delivery System for Specific Therapy of Acute Pneumonia. J. Control. Release.

[137] Stephan M.T., Moon J.J., Um S.H., Bershteyn A., Irvine D.J. (2010). Therapeutic Cell Engineering Using Surface-Conjugated Synthetic Nanoparticles. Nat. Med..

[138] Liu L., He H., Liu J. (2019). Advances on Non-Genetic Cell Membrane Engineering for Biomedical Applications. Polymers.

[139] Zaleski M.H., Chase L.S., Hood E.D., Wang Z., Nong J., Espy C.L., Zamora M.E., Wu J., Morrell L.J., Muzykantov V.R. (2025). Conjugation Chemistry Markedly Impacts Toxicity and Biodistribution of Targeted Nanoparticles, Mediated by Complement Activation. Adv. Mater..

[140] Hao M., Hou S., Li W., Li K., Xue L., Hu Q., Zhu L., Chen Y., Sun H., Ju C. (2020). Combination of Metabolic Intervention and T Cell Therapy Enhances Solid Tumor Immunotherapy. Sci. Transl. Med..

[141] Nie X., Liu Y., Song Y., Yao X., Chen Y., Lee H.-Y., Gao X. (2026). In Vivo Generation of CAR Myeloid Cells through Erythrocyte-Mediated mRNA Delivery for Cancer Immunotherapy. Sci. Transl. Med..

[142] Ponte J.F., Sun X., Yoder N.C., Fishkin N., Laleau R., Coccia J., Lanieri L., Bogalhas M., Wang L., Wilhelm S. (2016). Understanding How the Stability of the Thiol-Maleimide Linkage Impacts the Pharmacokinetics of Lysine-Linked Antibody-Maytansinoid Conjugates. Bioconjug. Chem..

[143] Wayteck L., Dewitte H., De Backer L., Breckpot K., Demeester J., De Smedt S.C., Raemdonck K. (2016). Hitchhiking Nanoparticles: Reversible Coupling of Lipid-Based Nanoparticles to Cytotoxic T Lymphocytes. Biomaterials.

[144] Chapanian R., Constantinescu I., Medvedev N., Scott M.D., Brooks D.E., Kizhakkedathu J.N. (2013). Therapeutic Cells via Functional Modification: Influence of Molecular Properties of Polymer Grafts on In Vivo Circulation, Clearance, Immunogenicity, and Antigen Protection. Biomacromolecules.

[145] Ji W., Smith P.N., Koepsel R.R., Andersen J.D., Baker S.L., Zhang L., Carmali S., Myerson J.W., Muzykantov V., Russell A.J. (2020). Erythrocytes as Carriers of Immunoglobulin-Based Therapeutics. Acta Biomater..

[146] Takayama Y., Kusamori K., Nishikawa M. (2019). Click Chemistry as a Tool for Cell Engineering and Drug Delivery. Molecules.

[147] Han J., Bhatta R., Liu Y., Bo Y., Elosegui-Artola A., Wang H. (2023). Metabolic Glycan Labeling Immobilizes Dendritic Cell Membrane and Enhances Antitumor Efficacy of Dendritic Cell Vaccine. Nat. Commun..

[148] Li X., Weller S., Clergeaud G., Andresen T.L. (2024). A Versatile Method for Conjugating Lipid Nanoparticles on T Cells through Combination of Click Chemistry and Metabolic Glycoengineering. Biotechnol. J..

[149] Resnier P., Lepeltier E., Emina A.L., Galopin N., Bejaud J., David S., Ballet C., Benvegnu T., Pecorari F., Chourpa I. (2019). Model Affitin and PEG Modifications onto siRNA Lipid Nanocapsules: Cell Uptake and In Vivo Biodistribution Improvements. RSC Adv..

[150] Feng C., Wang Y., Xu J., Zheng Y., Zhou W., Wang Y., Luo C. (2024). Precisely Tailoring Molecular Structure of Doxorubicin Prodrugs to Enable Stable Nanoassembly, Rapid Activation, and Potent Antitumor Effect. Pharmaceutics.

[151] Dong L., Ding J., Zhu L., Liu Y., Gao X., Zhou W. (2023). Copper Carbonate Nanoparticles as an Effective Biomineralized Carrier to Load Macromolecular Drugs for Multimodal Therapy. Chin. Chem. Lett..

[152] Li K., Yang J., Peng Y., Zheng H., Fu Z., Li M., Zhou W., Yu G. (2026). Advances in Metal Ion–Mediated Macromolecular Drug Delivery. Int. J. Pharm..

[153] Eweje F., Ibrahim V., Shajii A., Walsh M.L., Ahmad K., Alrefai A., Miyasato D., Davis J.R., Ham H., Li K. (2026). Self-Assembling Protein Nanoparticles for Cytosolic Delivery of Nucleic Acids and Proteins. Nat. Biotechnol..

[154] Sun Y., Lau S.Y., Lim Z.W., Chang S.C., Ghadessy F., Partridge A., Miserez A. (2022). Phase-Separating Peptides for Direct Cytosolic Delivery and Redox-Activated Release of Macromolecular Therapeutics. Nat. Chem..

[155] Wu H., Zhang X., Shu X., Zhang H., Zhou W., Zhang S., Luo C. (2026). Molecularly Tailored Artesunate Nanomedicine with Well-Balanced Nanoassembly and Anticancer Performance. Pharmaceutics.

[156] Wang H., Tang C., Zhang H., Guo L., Zou C., Zhang S., Liu J., Huang Q., Liu Y., Zhou W. (2025). A Biocompatible Tea Polyphenol Nanoplatform for Efficient Cytosolic Delivery of Protein Therapeutics. J. Control. Release.

[157] Goswami R., Jeon T., Nagaraj H., Zhai S., Rotello V.M. (2020). Accessing Intracellular Targets through Nanocarrier-Mediated Cytosolic Protein Delivery. Trends Pharmacol. Sci..

[158] Doshi N., Swiston A.J., Gilbert J.B., Alcaraz M.L., Cohen R.E., Rubner M.F., Mitragotri S. (2011). Cell-Based Drug Delivery Devices Using Phagocytosis-Resistant Backpacks. Adv. Mater..

[159] Zhu M.-H., Zhu X.-D., Long M., Lai X., Yuan Y., Huang Y., Zhang L., Gao Y., Shi J., Lu Q. (2023). Metal-Coordinated Adsorption of Nanoparticles to Macrophages for Targeted Cancer Therapy. Adv. Funct. Mater..

[160] França A., Aggarwal P., Barsov E.V., Kozlov S.V., Dobrovolskaia M.A., González-Fernández Á. (2011). Macrophage Scavenger Receptor A Mediates the Uptake of Gold Colloids by Macrophages In Vitro. Nanomed..

[161] Wang X., Meng X., Mao K., Chen H., Cong X., Liu F., Wang J., Liu S., Xin Y., Zhu G. (2023). Maleimide as the PEG End-Group Promotes Macrophage-Targeted Drug Delivery of PEGylated Nanoparticles In Vivo by Enhancing Interaction with Circulating Erythrocytes. Biomaterials.

[162] Lamoot A., Uvyn A., Kasmi S., De Geest B.G. (2021). Covalent Cell Surface Conjugation of Nanoparticles by a Combination of Metabolic Labeling and Click Chemistry. Angew. Chem..

[163] Mu Q., Yao K., Syeda M.Z., Zhang M., Cheng Q., Zhang Y., Sun R., Lu Y., Zhang H., Luo Z. (2023). Ligustrazine Nanoparticle Hitchhiking on Neutrophils for Enhanced Therapy of Cerebral Ischemia-Reperfusion Injury. Adv. Sci..

[164] Mullard A. (2020). Addressing Cancer’s Grand Challenges. Nat. Rev. Drug Discov..

[165] Wu J., Long Y., Li M., He Q. (2021). Emerging Nanomedicine-Based Therapeutics for Hematogenous Metastatic Cascade Inhibition: Interfering with the Crosstalk between “Seed and Soil”. Acta Pharm. Sin. B.

[166] Luo S., Feng J., Xiao L., Guo L., Deng L., Du Z., Xue Y., Song X., Sun X., Zhang Z. (2020). Targeting Self-Assembly Peptide for Inhibiting Breast Tumor Progression and Metastasis. Biomaterials.

[167] Zhu Y., Xue J., Chen W., Bai S., Zheng T., He C., Guo Z., Jiang M., Du G., Sun X. (2020). Albumin-Biomineralized Nanoparticles to Synergize Phototherapy and Immunotherapy against Melanoma. J. Control. Release Off. J. Control. Release Soc..

[168] Xu Y., Tang X., Guo Z., Wang Y., He C., Wei H., Xue Y., Ou Y., Ye L., Zhao Y. (2026). Biomineralized Nanoemulsion Delivers mRNA for Potent Cancer Immunotherapy. J. Control. Release Off. J. Control. Release Soc..

[169] Tong F., Wang Y., Xu Y., Zhou Y., He S., Du Y., Yang W., Lei T., Song Y., Gong T. (2024). MMP-2-Triggered, Mitochondria-Targeted PROTAC-PDT Therapy of Breast Cancer and Brain Metastases Inhibition. Nat. Commun..

[170] Kim S., Lee Y.K., Hong J.H., Park J., Choi Y., Lee D.U., Choi J., Sym S.J., Kim S., Khang D. (2018). Mutual Destruction of Deep Lung Tumor Tissues by Nanodrug-Conjugated Stealth Mesenchymal Stem Cells. Adv. Sci..

[171] Ning P., Du F., Wang H., Gong X., Xia Y., Zhang X., Deng H., Zhang R., Wang Z. (2024). Genetically Engineered Macrophages as Living Cell Drug Carriers for Targeted Cancer Therapy. J. Control. Release.

[172] Kong J., Deng Y., Xu Y., Zhang P., Li L., Huang Y. (2024). A Two-Pronged Delivery Strategy Disrupting Positive Feedback Loop of Neutrophil Extracellular Traps for Metastasis Suppression. ACS Nano.

[173] Deng C., Zhang Q., He P., Zhou B., He K., Sun X., Lei G., Gong T., Zhang Z. (2021). Targeted Apoptosis of Macrophages and Osteoclasts in Arthritic Joints Is Effective against Advanced Inflammatory Arthritis. Nat. Commun..

[174] Wang Q., Qin X., Fang J., Sun X. (2021). Nanomedicines for the Treatment of Rheumatoid Arthritis: State of Art and Potential Therapeutic Strategies. Acta Pharm. Sin. B.

[175] Li J., Long Y., Guo R., Ren K., Lu Z., Li M., Wang X., Li J., Wang Y., Zhang Z. (2021). Shield and Sword Nano-Soldiers Ameliorate Rheumatoid Arthritis by Multi-Stage Manipulation of Neutrophils. J. Control. Release.

[176] Gao Z., Wang N., Ma Y., Sun H., Li M., Dai Y., Jiang X., Ni S., Hao J., Cui J. (2024). Targeting Neutrophils Potentiates Hitchhiking Delivery of Drugs and Agonists for Postsurgical Chemo-Immunotherapy. Nano Today.

[177] Zhou H., He H., Liang R., Pan H., Chen Z., Deng G., Zhang S., Ma Y., Liu L., Cai L. (2021). In Situ Poly I:C Released from Living Cell Drug Nanocarriers for Macrophage-Mediated Antitumor Immunotherapy. Biomaterials.

[178] Pu Y., Duan Y., Li W., Lin H., Li Q., Yin B., Zhang K., Zhou B., Wu W. (2025). A Cerium Single-Atom Catalyst Enables Targeted Catalytic Therapy for Acute Kidney Injury via Neutrophil Hitchhiking. J. Control. Release.

[179] Gao H. (2016). Progress and Perspectives on Targeting Nanoparticles for Brain Drug Delivery. Acta Pharm. Sin. B.

[180] Shi S.M., Suh R.J., Shon D.J., Garcia F.J., Buff J.K., Atkins M., Li L., Lu N., Sun B., Luo J. (2025). Glycocalyx Dysregulation Impairs Blood-Brain Barrier in Ageing and Disease. Nature.

[181] Gheibihayat S.M., Cabezas R., Nikiforov N.G., Jamialahmadi T., Johnston T.P., Sahebkar A. (2021). CD47 in the Brain and Neurodegeneration: An Update on the Role in Neuroinflammatory Pathways. Molecules.

[182] Mac C.-H., Chiang Y.-W., Nguyen H.-N., Wang J.-T., Chu L.-A., Lin Y.-H., Nguyen V.K., Peng S.-Y., Lo S.-K., Chang Y. (2026). Chemically Programmed Prodrug Nanoparticles for Precise Oral Dopamine Delivery to Deep Brain Regions in Parkinson’s Disease. Adv. Mater..

[183] Bashor C.J., Hilton I.B., Bandukwala H., Smith D.M., Veiseh O. (2022). Engineering the next Generation of Cell-Based Therapeutics. Nat. Rev. Drug Discov..

[184] Mo F., Watanabe N., McKenna M.K., Hicks M.J., Srinivasan M., Gomes-Silva D., Atilla E., Smith T., Ataca Atilla P., Ma R. (2021). Engineered Off-the-Shelf Therapeutic T Cells Resist Host Immune Rejection. Nat. Biotechnol..

[185] Lipsitz Y.Y., Timmins N.E., Zandstra P.W. (2016). Quality Cell Therapy Manufacturing by Design. Nat. Biotechnol..

[186] Murray P.J., Allen J.E., Biswas S.K., Fisher E.A., Gilroy D.W., Goerdt S., Gordon S., Hamilton J.A., Ivashkiv L.B., Lawrence T. (2014). Macrophage Activation and Polarization: Nomenclature and Experimental Guidelines. Immunity.

[187] Stultz B.G., McGinnis K., Thompson E.E., Lo Surdo J.L., Bauer S.R., Hursh D.A. (2016). Chromosomal Stability of Mesenchymal Stromal Cells during In Vitro Culture. Cytotherapy.

[188] Jang T.H., Park S.C., Yang J.H., Kim J.Y., Seok J.H., Park U.S., Choi C.W., Lee S.R., Han J. (2017). Cryopreservation and Its Clinical Applications. Integr. Med. Res..

[189] Ekpo M.D., Xie J., Liu X., Onuku R., Boafo G.F., Tan S. (2022). Incorporating Cryopreservation Evaluations Into the Design of Cell-Based Drug Delivery Systems: An Opinion Paper. Front. Immunol..

[190] Zhang W., Wang M., Tang W., Wen R., Zhou S., Lee C., Wang H., Jiang W., Delahunty I.M., Zhen Z. (2018). Nanoparticle-Laden Macrophages for Tumor-Tropic Drug Delivery. Adv. Mater..

[191] Wang Y., Zhou C., Ding Y., Liu M., Tai Z., Jin Q., Yang Y., Li Z., Yang M., Gong W. (2021). Red Blood Cell-Hitchhiking Chitosan Nanoparticles for Prolonged Blood Circulation Time of Vitamin K1. Int. J. Pharm..

[192] Yang H., Yao L., Wang Y., Chen G., Chen H. (2023). Advancing Cell Surface Modification in Mammalian Cells with Synthetic Molecules. Chem. Sci..

[193] Szebeni J. (2014). Complement Activation-Related Pseudoallergy: A Stress Reaction in Blood Triggered by Nanomedicines and Biologicals. Mol. Immunol..

[194] Guo Q., Qian Z.-M. (2024). Macrophage Based Drug Delivery: Key Challenges and Strategies. Bioact. Mater..

[195] Lee Y., Lee S., Jon S. (2018). Biotinylated Bilirubin Nanoparticles as a Tumor Microenvironment-Responsive Drug Delivery System for Targeted Cancer Therapy. Adv. Sci..

[196] Peng S., Xiao F., Chen M., Gao H. (2022). Tumor-Microenvironment-Responsive Nanomedicine for Enhanced Cancer Immunotherapy. Adv. Sci..

[197] Son J., Parveen S., MacPherson D., Marciano Y., Huang R.H., Ulijn R.V. (2023). MMP-Responsive Nanomaterials. Biomater. Sci..

[198] Mao A.S., Özkale B., Shah N.J., Vining K.H., Descombes T., Zhang L., Tringides C.M., Wong S.-W., Shin J.-W., Scadden D.T. (2019). Programmable Microencapsulation for Enhanced Mesenchymal Stem Cell Persistence and Immunomodulation. Proc. Natl. Acad. Sci. USA.

